# Understanding the Physical and Molecular Basis of Stability of *Arabidopsis* DNA Pol λ under UV-B and High NaCl Stress

**DOI:** 10.1371/journal.pone.0133843

**Published:** 2015-07-31

**Authors:** Sujit Roy, Victor Banerjee, Kali Pada Das

**Affiliations:** Protein Chemistry laboratory, Department of Chemistry, Bose Institute, Kolkata, West Bengal, India; Russian Academy of Sciences, Institute for Biological Instrumentation, RUSSIAN FEDERATION

## Abstract

Here, we have investigated the physical and molecular basis of stability of *Arabidopsis* DNA Pol λ, the sole X family DNA polymerase member in plant genome, under UV-B and salinity stress in connection with the function of the N-terminal BRCT (breast cancer-associated C terminus) domain and Ser-Pro rich region in the regulation of the overall structure of this protein. Tryptophan fluorescence studies, fluorescence quenching and Bis-ANS binding experiments using purified recombinant full length Pol λ and its N-terminal deletion forms have revealed UV-B induced conformational change in BRCT domain deficient Pol λ. On the other hand, the highly conserved C-terminal catalytic core PolX domain maintained its tertiary folds under similar condition. Circular dichroism (CD) and fourier transform infrared (FT-IR) spectral studies have indicated appreciable change in the secondary structural elements in UV-B exposed BRCT domain deficient Pol λ. Increased thermodynamic stability of the C-terminal catalytic core domain suggested destabilizing effect of the N-terminal Ser-Pro rich region on the protein structure. Urea-induced equilibrium unfolding studies have revealed increased stability of Pol λ and its N-terminal deletion mutants at high NaCl concentration. *In vivo* aggregation studies using transient expression systems in *Arabidopsis* and tobacco indicated possible aggregation of Pol λ lacking the BRCT domain. Immunoprecipitation assays revealed interaction of Pol λ with the eukaryotic molecular chaperone HSP90, suggesting the possibility of regulation of Pol λ stability by HSP90 in plant cell. Overall, our results have provided one of the first comprehensive information on the biophysical characteristics of Pol λ and indicated the importance of both BRCT and Ser-Pro rich modules in regulating the stability of this protein under genotoxic stress in plants.

## Introduction

Several studies have indicated that plant growth and development under stress is largely influenced by the stability and activity of proteins which are induced and involved in environmental stress response. Therefore study of stability of such proteins and their response to abiotic and genotoxic stresses both under *in vitro* and *in vivo* conditions is important and has been the subject of extensive investigation towards understanding plant growth and developmental response under stress conditions over the last decades [1–4].

Repair of DNA damage is crucial to ensure the stability of the genome over generations [5]. DNA replication and repair synthesis in cells is carried out by DNA polymerases (DNA Pols) which are diverse both in number and function [6]. Among the four different families of DNA polymerases known in mammalian genome [7], family X DNA polymerase members are small, evolutionarily conserved relatively inaccurate enzymes involved in a number of DNA repair processes, including base excision repair (BER) and repair of double-strand breaks (DSBs) [8–9].

In recent years DNA Pol λ, a relatively newly identified X family member, creates special attention because of its involvement in some of the major DNA repair pathways in mammals. Similar to other mammalian family X members, DNA Pol λ is a relatively small, single-subunit enzyme lacking a 3’-5’ proofreading exonuclease activity and is widespread among higher eukaryotes, both in animals and plants [9]. The 575 amino acid long polypeptide of human Pol λ comprises an N-terminal BRCT domain, followed by a Ser-Pro (serine-proline) rich region and the C-terminal catalytic core PolX domain. The BRCT domain acts as the site for mediating protein-protein and protein-DNA interactions, while the Ser-Pro rich region has been indicated as the target site for the posttranslational modifications of Pol λ via phosphorylation for regulating the stability of the protein during progression of cell cycle [10]. The sequence of the 39 kDa catalytic core domain of human Pol λshares high degree of sequence similarity with human Pol β (33% amino acid identity), the major mammalian enzyme participates in BER. The catalytic core comprises of an N-terminal 8 kDa domain, unique to family X DNA Pols and a polymerase domain, organized in fingers, palm and the thumb sub-domains that are common to all polymerases.

Extensive studies have implicated crucial role of Pol λ in mammalian base excision repair, error-free translesion DNA synthesis in oxidative DNA damage [11–12] and repair of double strand breaks (DSBs) via non-homologous end joining (NHEJ) pathway [13–16]. In addition, recent studies have indicated specific requirement of Pol λ in cell cycle progression in response to oxidative damage and its functional link to the S phase specific DNA damage response machinery in cancer cells [17].

Analysis of rice (*Oryza sativa*) and *Arabidopsis thaliana* genome have demonstrated that Pol λ is the only member of family X DNA polymerase in plants and is encoded by single gene. Similar to human Pol λ, *Arabidopsis* DNA Pol λ (AtPolλ) comprises of two major domains, the N-terminal domain and the C-terminal highly conserved Pol X motif which is the active site for polymerase activity. The N-terminal part contains a nuclear localization signal (NLS), a BRCT module and a Ser–Pro-rich domain. *Arabidopsis* Pol λ shares ~39% identity and ~57% similarity in amino acid sequence with human Pol λ with a high degree of amino acid residue conservation at the Pol X domain, including the overall similarity in the sub-domain organization in the C-terminus PolX region which comprises of an N-terminal 8 kDa domain, unique to family X Pols and a polymerase domain, respectively.

Recent studies have established involvement of DNA Pol λ in multiple important DNA repair pathways in higher plants, including its role in gap-filling synthesis in BER [18], removal of UV-B induced photoproducts via nucleotide excision repair (NER) [19] and error-free translesion synthesis of oxidative DNA damage in *Arabidopsis* [20]. Our recent studies have demonstrated involvement of Pol λ in the repair of DSBs via NHEJ pathway in *Arabidopsis* [21].

Although earlier studies have indicated that Pol λ acts as an important component of nuclear DNA damage repair machinery in plant genome and that a stress tolerance trait has been implicated with Pol λ function, the structure-function properties, particularly the physical and molecular basis of stability of Pol λ in presence of abiotic and genotoxic stress in plant cell remains largely unknown. Therefore, in this study, we have systematically investigated the changes in the overall folding/unfolding characteristics, thermodynamic stability and changes in the secondary structural components of *Arabidopsis thaliana* DNA Pol λ (AtPolλ) following UV-B exposure and high NaCl treatment of the recombinant purified protein *in vitro*. Biophysical studies using purified recombinant full length and N-terminal deletion forms of Pol λ have indicated role of N-terminal BRCT domain and Ser-Pro rich region in regulating the stability of the protein. On the other hand, the C-terminal catalytic core PolX domain displayed greater stability as compared with the other parts of Pol λ. Analyses of the effects of UV-B light and high salinity on the stability of Pol λ *in vivo* using intact protoplasts from transiently transfected *Arabidopsis* and tobacco leaves indicated a higher possibility of aggregation of BRCT deficient Pol λ following UV-B stress. In addition, our results establish direct interaction of Pol λ with the molecular chaperone HSP90 in *Arabidopsis*, suggesting possible role of HSP90 in governing posttranslational regulation of Pol λ in plant genome.

## Materials and Methods

### Plant Materials, growth Conditions and treatments

*Arabidopsis* (*Arabidopsis thaliana*) Col-0 seeds were surface sterilized and grown in potting soil (Soilrite) or MS-agar by following the growth conditions described previously [19]. Surface sterilized tobacco seeds (*Nicotiana benthamiana*) were germinated on MS-agar media and two-weeks-old seedlings were then transferred in the potting soil and maintained in the growth chamber under 16-h-light/8-h-dark periods with a temperature regime of 25–28°C. The *atpolλ-1* and *atpolλ-3* mutant lines were described previously [21]. The *AtPolλ-C-TAP* (DKLAT1G10520.1) and *AtHSP90*.*1-C-TAP* (DKLAT5G52640) (pLIC6 plant expression vector expressing C-terminal-TAP-tag *AtPolλ* or *AtHSP90*.*1* under the CaMV-35S promoter) constructs were obtained from the ABRC, Ohio State University.

For the generation of transgenic *atpolλ-1* mutant lines expressing *Pol λ-Del 2* or *Pol λ-Del 3* cDNA fragments, the 1.26-kb *Pol λ-Del 2* and 996 bp *Pol λ-Del 3* cDNA fragments were amplified using the gene-specific primers (*Pol*λ-*Del2Fw*: 5’CGAGGATCCAAAATTGACTCAGAAGAAGTT3’ and *AtPolRev*: 5’GTTGAGCTCTCAGAGATTCCTCTCGTGT3’; *Pol*λ-*Del3Fw*: (5’ACGTGGATCCGATTTGAACAGAAATATCACTGA3’) and *AtPolRev*: 5’GTTGAGCTCTCAGAGATTCCTCTCGTGT3’) and cloned into the *Bam*HI-*Sac*I sites (underlined) of pCAMBIA 1201 binary vector under the control of the CaMV-35S constitutive promoter. Plants were transformed by *Agrobacterium* mediated floral dip method.

To examine the possibility of UV-B induced aggregation of DNA Pol λ, protoplasts isolated from leaf mesophyll cells of 25-28-days-old wild-type *Arabidopsis*, transgenic *atpolλ-1* ^*Polλ-Del2ox*^, *atpolλ-1* ^*Polλ-Del3ox*^ seedlings (*atpolλ-1* null mutant seedlings expressing either *Polλ-Del2* or *Polλ-Del3* cDNA under the CaMV-35S constitutive promoter) and tobacco (*Nicotiana benthamiana*) leaves (transiently expressing full length C terminal TAP *AtPolλ* under the constitutive 35S CaMV promoter in pLIC6 plant expression vector) were irradiated with UV-B light following the method described previously [22–23] with some modifications (S1 File).

### Protoplast Preparation from *Arabidopsis* and tobacco leaf mesophyll cells and infiltration of tobacco leaves with *Agrobacteria*

Protoplast isolation from *Arabidopsis* and tobacco (*Nicotiana benthamiana*) leaves were carried out following Salvucci and Anderson (1987) and Kim and Somers (2010) with minor modifications [24–25] (S1 File). For *in planta* analysis of the possibility of aggregation of Pol λ in presence of salinity stress, AtPolλ protein was transiently expressed in tobacco (*Nicotiana benthamiana*) leaves by infiltration of tobacco leaves with *Agrobacteria* following the procedure described previously [26] (S1 File).

### Protein analysis

Details of protein extraction, immunoblot procedures, immunoprecipitation and co-immunoprecipitation are provided in S1 File.

### Expression, and Purification of recombinant full length and N terminal deletion fragments of Polλ

The molecular cloning and expression of full length recombinant *Arabidopsis thaliana* DNA polymerase λ (*AtPolλ)* and its N-terminal deletion constructs in *E coli* cells have been described previously [21]. Details of the purification techniques of the recombinant proteins are provided in S1 File.

### UV-B irradiation and salt treatment of recombinant Pol λ

The UV-B irradiation experiments were performed in a 1 cm light-path quartz cuvette and following the method described previously [27–28] with some modifications. ~500 μl protein samples (stored at -80°C) were brought to room temperature (25°C) ~1 h before UV-treatment, then centrifuged briefly at 5000 rpm for 5 min at 25°C and the supernatant was irradiated with an UV-B dose of 200 J/m^2^ for different time points up to 4 h in the dark at 25°C. Precautions were taken so that temperature variation of protein samples during UV irradiation did not exceed 1–2°C. UV-B dose in the range between 250–300 J/m^2^ and beyond produced severe photo chromatic effects, and therefore UV-B dose beyond 200 J/m^2^ was avoided. For salt treatment, the purified recombinant protein samples were incubated in presence of different concentrations of NaCl for 4 h or various time points at room temperature (25°C).

### Tryptophan Fluorescence Spectroscopy

Tryptophan fluorescence spectra were measured in a Jasco Spectrofluorometer FP—8500. Control or treated protein sample (0.05 mg/mL purified protein in a final volume of 600 μL of 50 mM Tric-HCl buffer, pH 7.5 containing 1 mM β-ME and 1 mM PMSF) was taken in a quartz cuvette (4 X 4 mm). Tryptophan fluorescence emission spectra were scanned between 300–400 nm using excitation wavelength of 295 nm. Both excitation and emission slits were set at 5 nm. Each spectrum was an average of three scans. Respective control buffer (no protein) spectrum was subtracted from sample spectrum to generate the fluorescence spectrum of the protein. UV-B mediated oxidation of tryptophan residues to N-formylkynurenine was measured using fluorometry with excitation wavelength of 365 nm and the emission wavelengths ranging from 390 to 530 nm, respectively [27].

### Acrylamide and Iodide Fluorescence Quenching assays

Tryptophan fluorescence quenching assay was carried out by titration of protein sample (untreated control or treated) with 5 M freshly prepared acrylamide or potassium iodide solution, in 50 mM Tric-HCl buffer, pH 7.5 containing 1 mM β-ME and 1 mM PMSF. 2.0 ml of 0.05 mg/ml protein solution was taken in a 3-ml quartz cuvette. Small aliquots of freshly prepared 5 M acrylamide or potassium iodide (quencher) was added to the protein sample in the cuvette, after each addition solution was mixed by gentle pipetting and left to attain equilibrium for 2 min and then the emission spectrum was recorded. The fluorescence spectral readings for all concentrations of the titrant were corrected for the “dilution effect” because of addition of the titrant. The spectral readings were also corrected for the ‘inner filter effect’ following Eq 1:

$${\text{F}}_{\text{corr}}\;=\text{ F }*\text{ antilog}\left[\left({\text{A}}_{\text{ex}}\;+{\text{ A}}_{\text{em}}\right)/2\right]$$ (1)

Here, F and F_corr_ stand for the uncorrected and corrected fluorescence, while A_ex_ and A_em_ indicate the absorbance of the solution at the excitation and emission wavelengths, respectively. The quenching data were analyzed by using the Stern-Volmer plot (Eq 2).

$${\text{F}}_{\text{o}}\;/\text{F }=\;1\;+{\text{ K}}_{\text{SV}}\left[\text{Q}\right]$$ (2)

where F_o_ and F indicate the fluorescence intensities in absence and presence of the quencher and [Q] indicates the molar concentration of the quencher. A plot of F_o_ /F versus [Q] yields a straight line with a slope of K_SV_, called the Stern-Volmer constant.

### Bis-ANS binding analysis

Bis-ANS (4,4’-dianilino-1,1’-binaphthyl-5,5- disulfonic acid) binding study was carried out by following the method described previously [29]. Purified recombinant full length Pol λ or each of the N-terminal deletion fragment (0.02 mg/ml, either untreated control or 4 h UV-B irradiated or incubated in presence of 500 mM NaCl for 2 h at 25°C) was taken in a 3 ml quartz cuvette. The fluorimeter cuvette was placed inside a Jasco Spectrofluorometer FP—8500 and the sample was titrated with an aqueous solution of 300 μM Bis-ANS by adding a small aliquot at a time. After each addition, the solution was mixed by gentle pipetting and left for 2 min to attain equilibrium. The fluorescence emission spectrum was then recorded between 450 and 550 nm by using an excitation wavelength of 390 nm. For analyzing the data according to the Scatchard equation, a titration of 0.2 μM Bis-ANS using 10.0 mg/ml or 4.0 mg/ml of recombinant protein was performed. The reverse titration data were used to obtain the quantitative relationship between fluorescence intensity change and bound Bis-ANS [30].

### Urea Unfolding assay

Equilibrium urea unfolding experiments were carried out to assess the thermodynamic stability of the purified recombinant full length and N-terminal deletion fragments (Del2 and Del3) of Pol λ under stress conditions (UV-B and salt treatment) at 25°C by following the method described previously [31] with some modifications. 0.05 mg/ml of purified protein sample (untreated control, UV-B irradiated for 4 h or pre-incubated in presence of 500 mM NaCl for 2 h at room temperature), was incubated in presence of 0–8 M urea at room temperature (25°C) for 16 h. Tryptophan fluorescence spectra of the samples were scanned from 300–400 nm using excitation wavelength of 295 nm. Corresponding control buffer spectra were subtracted from each sample spectra for each set of assay to generate the fluorescence spectra of the protein samples at different urea concentrations. The unfolding profiles were constructed by plotting the intensity ratio at 337 and 350 nm (I_337_/I_350_) as a function of urea concentration. The urea unfolding profiles were further analyzed according to three-state transition, and the free energy of unfolding was determined by employing a global fit [29, 31]. The experimental data were fitted to the following equation using Microcal Origin 6.0 software:

$$F(U)=\frac{I_{0}+I_{1}\text{exp}[(-\Delta G{{}^{\circ}}_{NI}+M_{NI}[U])/RT]+I_{\infty }\text{exp}[(-\Delta G{{}^{\circ}}_{IU}+M_{IU}[U])/RT]}{I_{0}+\text{exp}[(-\Delta G{{}^{\circ}}_{NI}+M_{NI}[U])/RT]+\text{exp}[(-\Delta G{{}^{\circ}}_{IU}+M_{IU}[U])/RT]}$$

Here I_0_, I_1_, and I_∞_ represent the fluorescence signal intensities for 100% native (at 0 M urea), proposed intermediate and unfolded (at 8 M urea) form respectively. ΔG^0^_NI_, ΔG^0^_IU_ and ΔG^0^ refer to the standard free energy change between native and intermediate, intermediate and unfolded, and native and unfolded forms, respectively.

### Circular dichroism (CD) spectroscopy

CD spectroscopy of control, UV-B irradiated and high salt treated purified recombinant protein samples were performed by following the protocol described previously [32–33] with some modifications. Protein samples were dialyzed separately against 5 mM sodium phosphate buffer, pH 7.5, overnight at 4°C with three changes. Far-UV CD spectroscopy was carried out with ~0.5 mg/ml purified recombinant full length Pol λ, Pol λ-Del2 and Pol λ-Del3 proteins in a total 1.5 mL reaction volume. Protein samples were UV-B irradiated with a dose of ~200 J/m^2^ for 4 h at 25°C or pre-incubated in presence of 500 mM NaCl for 2 h at 25°C. The far-UV CD spectra was recorded at 25°C in the wavelength range of 200–260 nm in a JASCO-810 Spectropolarimeter using a 1 mm path-length rectangular cell. Five scans were recorded at 0.5 data pitch with 20 nm/min scan speed, 4.0 nm band width and 1 s time constant respectively. All spectra were buffer corrected. The CD results were expressed in terms of milideg (θ).

### Fourier transform infrared (FT-IR) spectroscopy

FT-IR spectroscopy was carried out by following the method described previously [31, 34] with some modifications. For FT-IR measurement, 50 μl of ~7.5 μg of purified recombinant protein sample (full length or deletion versions of Pol λ) was taken in a microcon filter device fitted with a 3-kDa cut-off membrane. The protein sample was diluted with ~200 μl D_2_O (Sigma). The sample was then centrifuged at 14000 rpm for 8 to 10 min at 4°C until the sample volume reached ~50 μl. It was further diluted with ~200 μl D_2_O and again centrifuged. This D_2_O exchange process was repeated 3–4 times. Finally, the D_2_O exchanged protein sample (10 μl) was carefully placed in between two clean CaF_2_ windows separated by a 50 μm thick teflon spacer. FT-IR scans were collected in the range of 1600–1700 cm^-1^ at the resolution of 2 cm^-1^ using a Spectrum 100 FT-IR spectrometer (Perkin Elmer). Spectrum of D_2_O containing buffer was subtracted from each sample spectrum. Fourier self-deconvolution was used to resolve the peak positions in the broad amide I contour. Curve fitting of the original amide I contours was performed using Thermo GRAMS AI software [31, 34]. The percentage of each secondary structure was calculated by dividing the area of the corresponding peak with the total area of the amide I contour.

### Light scattering measurements for *in vitro* protein aggregation studies

UV-B and salinity induced aggregation of purified recombinant proteins (0.1 mg/ml) was monitored by measuring static light scattering using a spectrophotometer (Shimadzu UVPC v 3.9) [29, 31]. Reactions were carried out in a 1 cm light-path black masked quartz microcuvette in a total volume of 500 μL at room temperature (25°C). Apparent O.D. of the samples was measured at 360 nm over a period of 240 min.

## Results

### Tryptophan fluorescence studies of recombinant DNA Pol λ under genotoxic stress

Previously we have demonstrated that UV-B irradiation enhances the expression of Pol λ in *Arabidopsis* seedlings and reported its involvement in repair of UV-B induced DNA damage via nucleotide excision repair pathway [19]. In addition, our recent findings have established role of Pol λ in repair of high salinity induced DNA double strand breaks (DSBs) in *Arabidopsis* [21]. Based on this information, we were next interested to specifically examine the effects of genotoxic stress like UV-B irradiation and high salinity on the structural properties of Pol λ to understand the stability of this important nuclear DNA damage repair protein under genotoxic stress in plant genome. To address this issue, we first studied the tryptophan fluorescence spectra of the bacterially expressed purified recombinant full length *Arabidopsis* DNA Pol λ (wild-type) under control condition and after UV-B exposure or in presence of added salt to monitor whether the protein undergoes any conformational changes under such stress conditions. We have also utilized three other recombinant purified distinct N-terminal deletion mutants of this protein, lacking the N-terminal NLS sequence (Del 1), the BRCT module (Del 2) and the Ser/Pro rich (S-P) domain (Del 3) respectively, in the similar set of experiments to understand the relative influence of the N-terminal domains on the conformational stability of the highly conserved C-terminal catalytic core domain (PolX region, Del 3) and the full length protein as a whole (S1 Fig).

**Fig 1:**
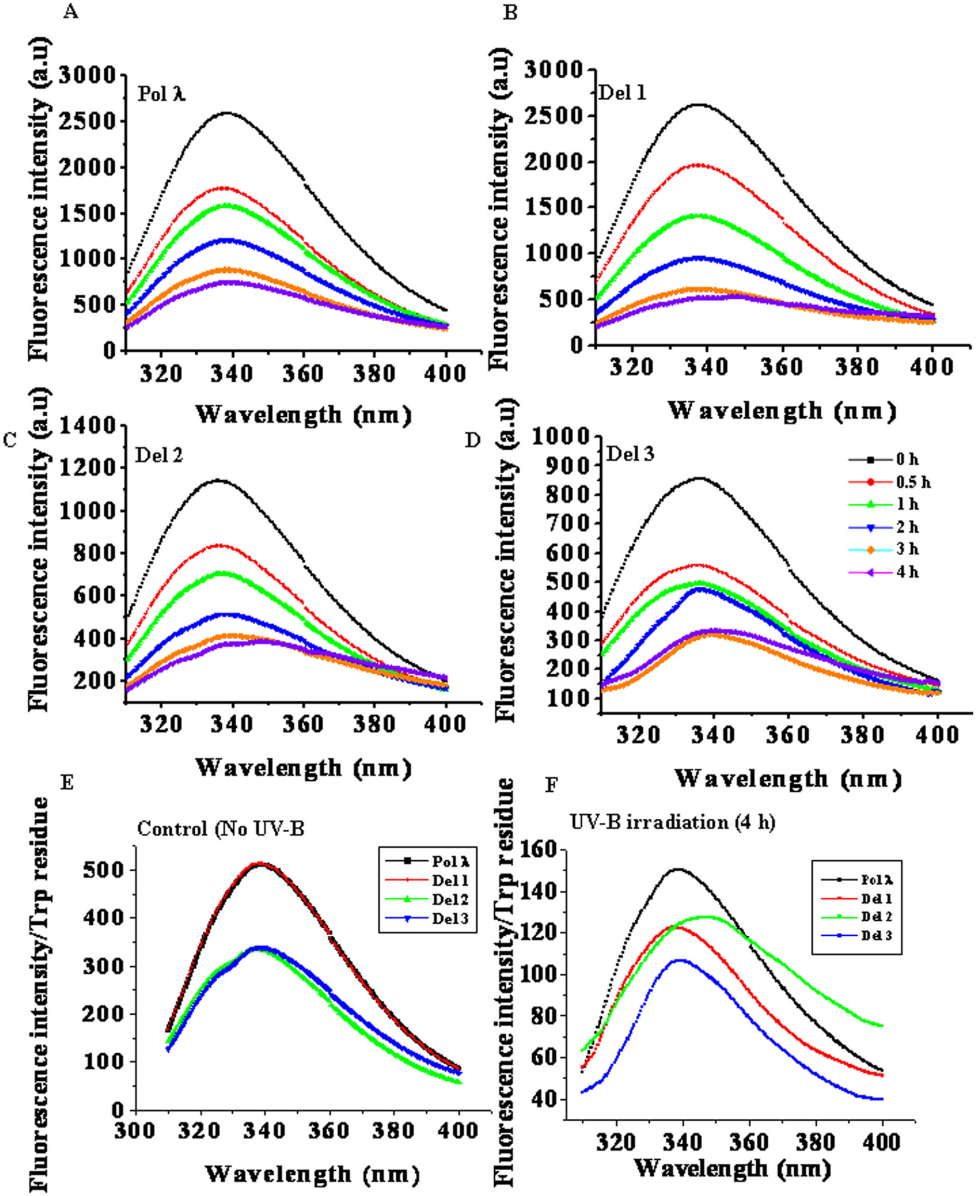
Tryptophan fluorescence spectra of UV-B irradiated purified recombinant full length and N terminus truncated forms of *Arabidopsis* DNA Pol λ (AtPolλ). 0.05 mg/mL of purified (A) full length recombinant Pol λ, (B) Del 1, (C) Del 2, and (D) Del 3 protein samples in a final volume of 600 μL of 50 mM Tric-HCl buffer, pH 7.5 (containing 1 mM β-ME and 1 mM PMSF) were irradiated with an UV-B dose of ~200 J/m^2^ for the indicated time points in the dark at 25°C. Tryptophan fluorescence spectra of control and UV-B irradiated protein samples were measured using excitation wavelength of 295 nm. The emission wavelengths were set in the range between 300 to 400 nm with the emission scan speed of 240 nm/min. The 0-h time point served as the control. (E) Normalized tryptophan fluorescence intensity per tryptophan residue in full length recombinant Pol λ, Del 1, Del 2 and Del 3 fragments under control condition (without UV-B exposure). (F) Normalized tryptophan fluorescence intensity per tryptophan residue in full length recombinant Pol λ, Del 1, Del 2 and Del 3 fragments after 4 h of UV-B (~200 J/m^2^) at room temperature.

In case of UV-B stress, recombinant purified protein samples were exposed to UV-B light (200 J/m^2^) for up to 4 h as described under ‘Materials and Methods’. As shown in Fig 1, with increasing time of exposure to UV-B radiation, the tryptophan fluorescence intensity gradually decreased in both full length Pol λ and its N-terminal deletion mutant proteins. The emission maxima in almost all cases remained nearly constant at around 340 nm indicating that the polarity of the tryptophan microenvironment remained largely unaltered (Fig 1A–1D). We reasoned that the change in the tryptophan fluorescence intensity following UV-B exposure could either be due to conformational changes within the protein molecule or as a result of degradation of tryptophan residues or due to both. At this point, the analysis of the change in fluorescence intensities of the protein samples became slightly complicated as the full length Pol λ and the N-terminal mutants have different number of tryptophan residues—the full length Pol λ and Del 1 have 5 each while Del 2 and Del 3 have three tryptophan residues each, respectively (S1B Fig). Therefore, in order to compare the difference in tryptophan fluorescence intensities of full length Pol λ and the other N-terminal deletion mutants, we used normalized tryptophan fluorescence intensity per tryptophan residue for control and UV-B irradiated proteins (Fig 1E and 1F). Under control condition (without UV-B exposure), full length Pol λ showed the maximum intensity with emission maximum at 340 nm (Fig 1E). Elimination of the nuclear localization signal residues (Del 1) caused no significant change in the spectrum, indicating that the signal residues have no prominent effect on the microenvironment of the tryptophan residues. On the other hand, further elimination of the BRCT domain (Del 2) led to an appreciable decrease in the normalized fluorescence intensity (Fig 1E), while the elimination of the additional S-P domain from Del 2 had no further influence on the tryptophan spectrum in Del 3, suggesting that the two tryptophan residues in the BRCT domain had higher contribution to tryptophan fluorescence intensity than the rest of the three tryptophan residues which are located within the C-terminal PolX domain (Del 3) and therefore, the S-P region does not contribute to the tryptophan microenvironment of the protein. However interesting results were obtained for UV-B irradiated proteins (Fig 1F). UV-B irradiation for 4 hr drastically reduced normalized fluorescence intensity of full length Pol λ as well as the N-terminal deletion mutants. Whereas Del 1 showed λ_max_ around 340 nm, Del 2 not only showed reduced fluorescence intensity, but also displayed a clear shift of λ_max_ to 350 nm, indicating exposure of tryptophan residues. Del 3 had reduced intensity than Del 2 (Fig 1F). Since the protein variants of our interest have different number of tryptophan residues, we have used normalized plot of I_337_/ I_350_ with the time of UV-B exposure to monitor the UV-B induced conformational change of the protein variants (S4 Fig). Though, Pol λ and Del 1 did not show any appreciable change in the intensity ratio, both Del 2 and Del 3 showed steady decrease in I_337_/ I_350_ value with the increase in time of irradiation. However, change in the ratios of I_337_/ I_350_ for Del 2 and Del 3 were found to be ~18% and ~12%, respectively, suggesting that removal of the BRCT domain (Del 2) caused Pol λ to become structurally more vulnerable towards UV-B irradiation compared to the catalytic core domain (Del 3). Taken together, these results indicate that UV-B exposure helped expose primarily the tryptophan residues located within the catalytic core PolX domain (Del 3) of Del 2 fragment of Pol λ, resulting in the shift in λ_max_ after UV-B exposure, while the presence of Ser-Pro rich domain in Del 2 probably reduces fluorescence quenching after UV-B exposure.

**Fig 2:**
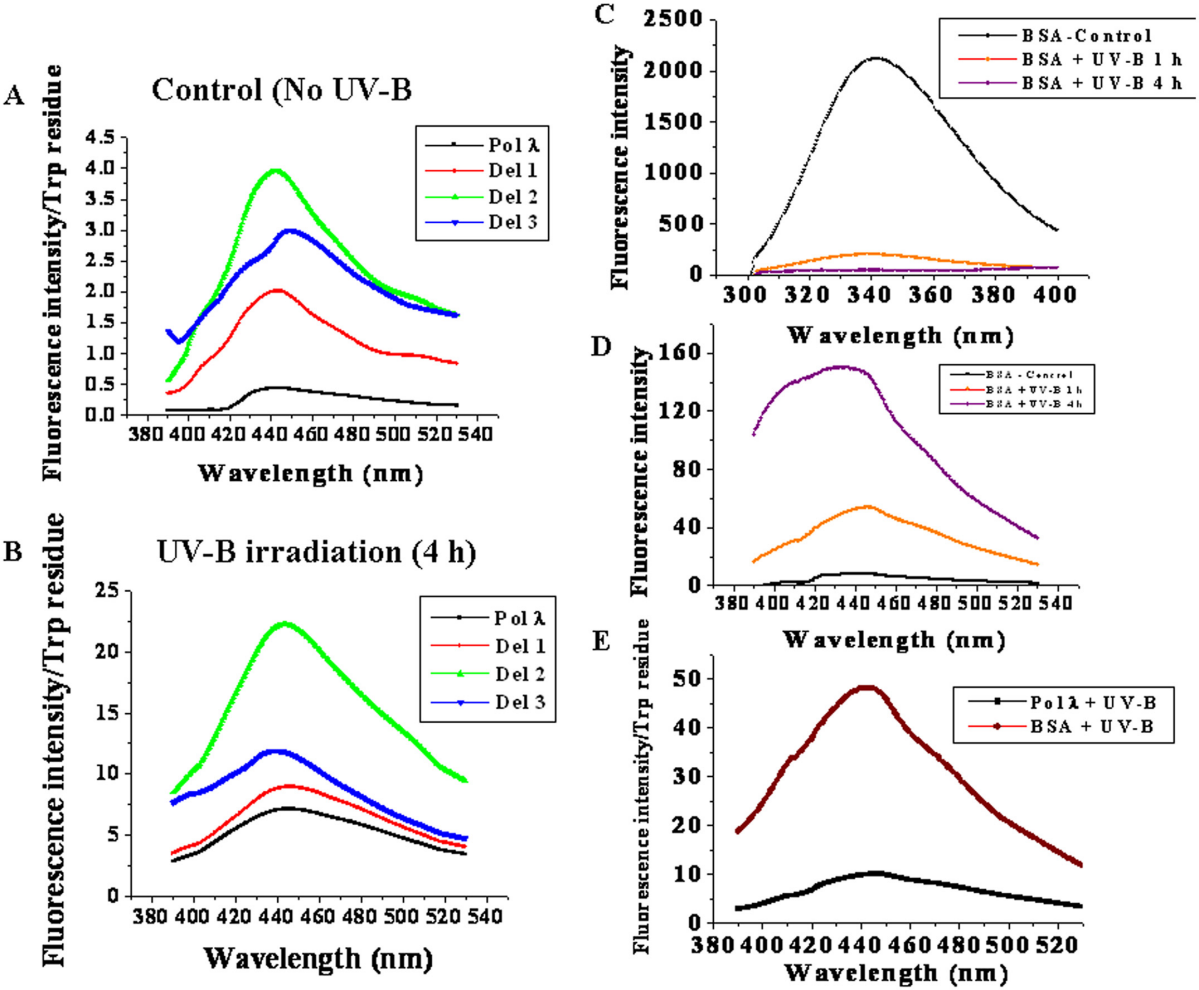
UV-B mediated oxidative degradation of tryptophan residues to *N*-formylkynurenine in Pol λ and its N-terminal deletion mutants. (A) Normalized tryptophan fluorescence intensity per tryptophan residue in untreated control or (B) 4 h of UV-B exposed full length recombinant Pol λ, Del 1, Del 2 and Del 3 fragments. Fluorescence spectra were measured using excitation wavelength of 365 nm with the emission wavelengths ranging from 390 to 530 nm and emission scan speed of 240 nm/min, using 0.05 mg/mL of purified protein samples for analyzing the UV-B induced oxidation of tryptophan to *N*-formylkynurenine. (C) Tryptophan fluorescence spectra of 0.05 mg/ml of BSA (Sigma fraction V) under control or after 1 and 4 h of UV-B exposure. (D) Fluorescence spectra of control and UV-B BSA were measured using excitation wavelength of 365 nm with the emission wavelengths ranging from 390 to 530 nm to monitor the UV-B induced oxidation of tryptophan to *N*-formylkynurenine. (E) Normalized tryptophan fluorescence intensity per tryptophan residue in 4 h of UV-B exposed full length recombinant Pol λ and BSA. Fluorescence spectra were measured using excitation wavelength of 365 nm with the emission wavelengths ranging from 390 to 530 nm to compare the UV-B induced oxidation of tryptophan to *N*-formylkynurenine in Pol λ and BSA.

It is known that UV-B exposure causes degradation of tryptophan residues to *N*-formylkynurenine which shows characteristic tryptophan emission spectrum with maximum at around 440 nm [27], resulting in decreased tryptophan fluorescence intensity. In order to assess this possibility, we next compared the *N*-formylkynurenine spectra of control with the 4 hr UV-B exposed protein samples (Fig 2A and 2B). As shown in Fig 2A, the control proteins produced much less amount of *N*-formylkynurenine as compared to the UV-B exposed ones (Fig 2B). *N*-formylkynurenine in control samples might have generated from the UV-B exposure of the samples in the fluorescence spectrometer during the experiment. It was found that full length Pol λ produced the least amount of *N*-formylkynurenine compared to its N-terminal deletion mutants in terms of both total fluorescence (not shown) and normalized fluorescence spectra (Fig 2A). Among the deletion mutants, *N*-formylkynurenine formation was maximum in Del 2 in both control and after UV-B exposure (Fig 2A and 2B). It appeared that deletion of the signal domain (Del 1) did not significantly affect tryptophan degradation by UV-B, while removal of the BRCT domain from Pol λ (Del 2) caused higher exposure of the tryptophan residues in the C-terminal catalytic core PolX domain (Del 3 fragment) for UV-B mediated damage. Interestingly, the UV-B mediated tryptophan damage was somewhat controlled by further removal of the S-P region from Del 2 (Del 3). We also compared the UV-B induced damage of tryptophan residues of Pol λ with BSA, which has two tryptophan residues (Trp 134, Trp 214), as control. In contrast to Pol λ, UV-B exposure drastically reduced the tryptophan fluorescence intensity in BSA (Figs 1A and 2C). In addition, after 4 h of UV-B exposure, degradation of tryptophan to *N*-formylkynurenine was much higher in BSA (~5-times more) than in full length Pol λ (Fig 2D and 2E). Taken together, these results indicate that Pol λ possesses an intrinsic UV-B tolerance property. However, removal of the BRCT domain increased the rate of UV-B mediated damage, while further deletion of the S-P region again stabilized the C-terminal catalytic core PolX domain (Del 3) of Pol λ under UV-B stress, indicating that the S-P region has a destabilizing effect on the structure of Del 2 fragment of Pol λ.

**Fig 3:**
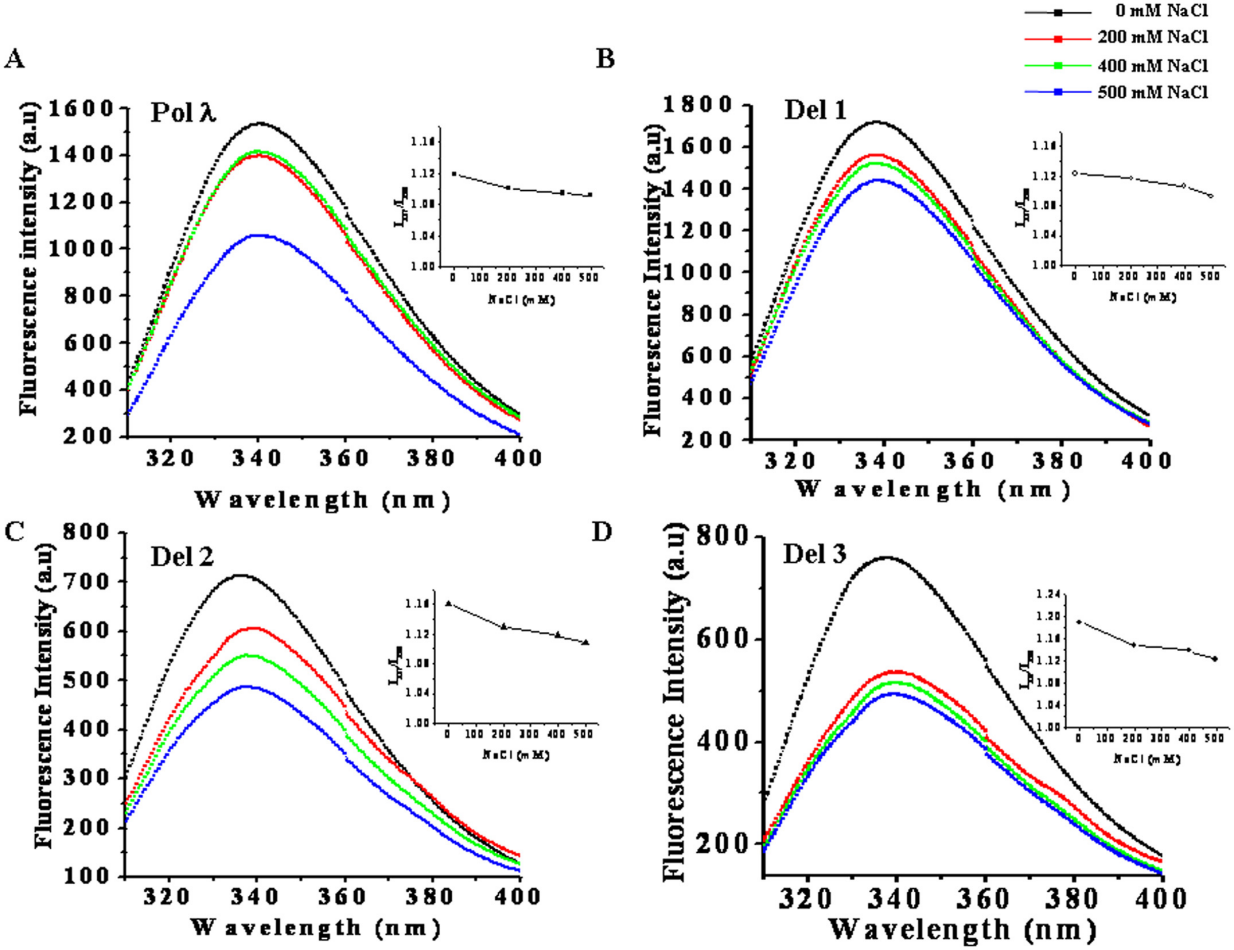
Tryptophan fluorescence spectra of high salt treated purified recombinant full length and N terminus deletion versions of Pol λ. 0.05 mg/mL of purified (A) full length recombinant Pol λ, (B) Del 1, (C) Del 2, and (D) Del 3 protein samples in a final volume of 600 μL of 50 mM Tric-HCl buffer, pH 7.5 (containing 1 mM β-ME and 1 mM PMSF) were subjected to increasing concentrations of NaCl treatment for 2 h at 25°C. Tryptophan fluorescence spectra of protein samples were measured using excitation wavelength of 295 nm. The emission wavelengths were set in the range between 300 to 400 nm with the emission scan speed of 240 nm/min. Inset images indicate ratio of fluorescence intensity at 337 nm to the same at 350 nm plotted as function of NaCl concentration.

The tryptophan fluorescence intensity of full length Pol λ and its N-terminal deletion mutants also decreased in presence of increasing concentrations of NaCl (Fig 3) as well as increasing time of exposure to high NaCl concentrations (S5 and S6 Figs). Full length Pol λ showed only marginal decline in tryptophan fluorescence intensity when NaCl concentration was increased to 400 mM. However at 500 mM NaCl, ~35% drop in intensity was observed as compared to control condition (Fig 3A). The Del 1 mutant showed gradual decrease in tryptophan fluorescence intensity with increasing NaCl concentration but the decrease was only ~ 15% at 500 mM NaCl (Fig 3B). Del 2 behaved in the same way as Del 1, but the decrease in tryptophan fluorescence intensity was ~ 35% in presence of 500 mM NaCl (Fig 3C). Tryptophan fluorescence intensity of Del 3 protein quenched effectively in presence of increasing NaCl concentrations and ~35% drop in intensity was observed at 200 mM NaCl. However, it was also interesting to note that at 500 mM NaCl, the normalized tryptophan fluorescence intensities (intensity per tryptophan residue) in both Del 2 and Del 3 were comparable (data not shown).

### Tryptophan fluorescence quenching analysis

To further substantiate the tryptophan fluorescence spectral pattern of full length recombinant Pol λ and the N-terminal deletion mutants, we next performed tryptophan fluorescence quenching assays using untreated control and UV-B or salt treated protein samples. Tryptophan quenching experiments were carried out using acrylamide and iodide as the complementary set of water soluble quenchers. Acrylamide, being a neutral quencher, has the greater ability to penetrate into the protein interior to quench buried tryptophan residues. In contrast, iodide, a negatively charged and highly hydrated bulky quencher, has the less ability to enter the protein interior. Thus, in case of iodide, the quenching ability is mainly related to the tryptophan residues located near the surface of the protein [35]. The tryptophan fluorescence quenching data were analysed by Stern-Volmer plot considering variable and heterogeneous emission from the different tryptophan residues. The Stern-Volmer constant, K_SV_ has been considered as the quenching constant, representing the weighted average of the quenching constants of individual tryptophan residues and may include contribution from both static and dynamic quenching, respectively.

The acrylamide and iodide quenching data for recombinant full length Pol λ and its N-terminal deletion mutants under control condition and after UV-B or high NaCl stress have been shown in S7 Fig as Stern-Volmer plot. The values of K_SV_ in the indicated conditions are summarized in Table 1. For acrylamide quenching, full length Pol λ (untreated control) showed the K_SV_ value of 4.7 M^-1^. After UV-B exposure or 500 mM NaCl treatment, K_SV_ of Pol λ was increased to 5.5 M^-1^ and 5.7 M^-1^, indicating relatively higher exposure of tryptophan residues following stress. On the other hand, for iodide quenching of full length Pol λ, as compared to control (K_SV_ 5.2 M^-1^), UV-B exposure reduced the K_SV_ value (4.4 M^-1^), while 500 mM NaCl treatment did not significantly alter the K_SV_ (5.5 M^-1^). The Del 1 fragment, with similar tryptophan microenvironment like Pol λ, did not show any significant difference from Pol λ in the K_SV_ values of acrylamide or iodide quenching of untreated control or treated protein (data not shown). For acrylamide quenching of Del 2 fragment of Pol λ, particularly after UV-B exposure, the K_SV_ value was found to increase from 5.7 M^-1^ (control) to 7.0 M^-1^, while in presence of 500 mM NaCl, the K_SV_ for Del 2 (5.2 M^-1^) did not show any prominent change than control. More or less similar pattern was obtained for iodide quenching in Del 2 fragment under control and after UV-B or salt treatment, respectively (Table 1). For both acrylamide and iodide quenching, Del 3 protein showed relatively higher accessibility of tryptophans to the quencher after UV-B exposure (K_SV_ values of 6.5 M^-1^ and 5.8 M^-1^ for acrylamide and iodide, respectively) and salt treatment (K_SV_ 6.0 M^-1^ and 5.3 M^-1^ for acrylamide and iodide, respectively) than untreated control (K_SV_ values of 5.3 M^-1^ and 4.9 M^-1^ for acrylmide and iodide, respectively). Taken together, these results indicate marginal conformational change in full length Pol λ following the indicated doses of UV-B exposure or in presence of higher NaCl concentration used in this study. However, appreciable change in the microenvironment of tryptophan residues in UV-B irradiated Del 2 protein caused relatively easier penetration of the quencher molecule. These results were also consistent with the pattern of the tryptophan fluorescence spectra of the proteins in general (Figs 1 and 3).

**Table 1:** Acrylamide and KI quenching constants (K_SV_) of control, UV-B irradiated and high salt treated purified recombinant full length and N terminus deletion versions of recombinant purified *Arabidopsis thaliana* DNA Pol λ.

	Acrylamide	Iodide
System	K_SV_ (M^-1^)	K_SV_ (M^-1^)
Pol λ (Control)	4.7 ± 0.2	5.2 ± 0.2
Pol λ + 200 J/m^2^ UV-B	5.5 ± 0.4	4.4 ± 0.2
Pol λ + 500 mM NaCl	5.7 ± 0.2	5.5 ± 0.3
Pol λ-Del2 (Control)	5.7 ± 0.4	5.7 ± 0.4
Pol λ-Del2 + 200 J/m^2^ UV-B	7.0 ± 0.3	7.2 ± 0.5
Pol λ-Del2 + 500 mM NaCl	5.2 ± 0.4	5.2 ± 0.6
Pol λ-Del3 (Control)	5.3 ± 0.5	4.9 ± 0.2
Pol λ-Del3 +200 J/m^2^ UV-B	6.5 ± 0.6	5.8 ± 0.9
Pol λ-Del3 + 500 mM NaCl	6.0 ± 0.4	5.3 ± 0.1

# (Errors represent SD from three independent measurements)

### Conformational change of recombinant full length Pol λ and its N-terminal deletion mutants after UV-B and salinity stress

**Fig 4:**
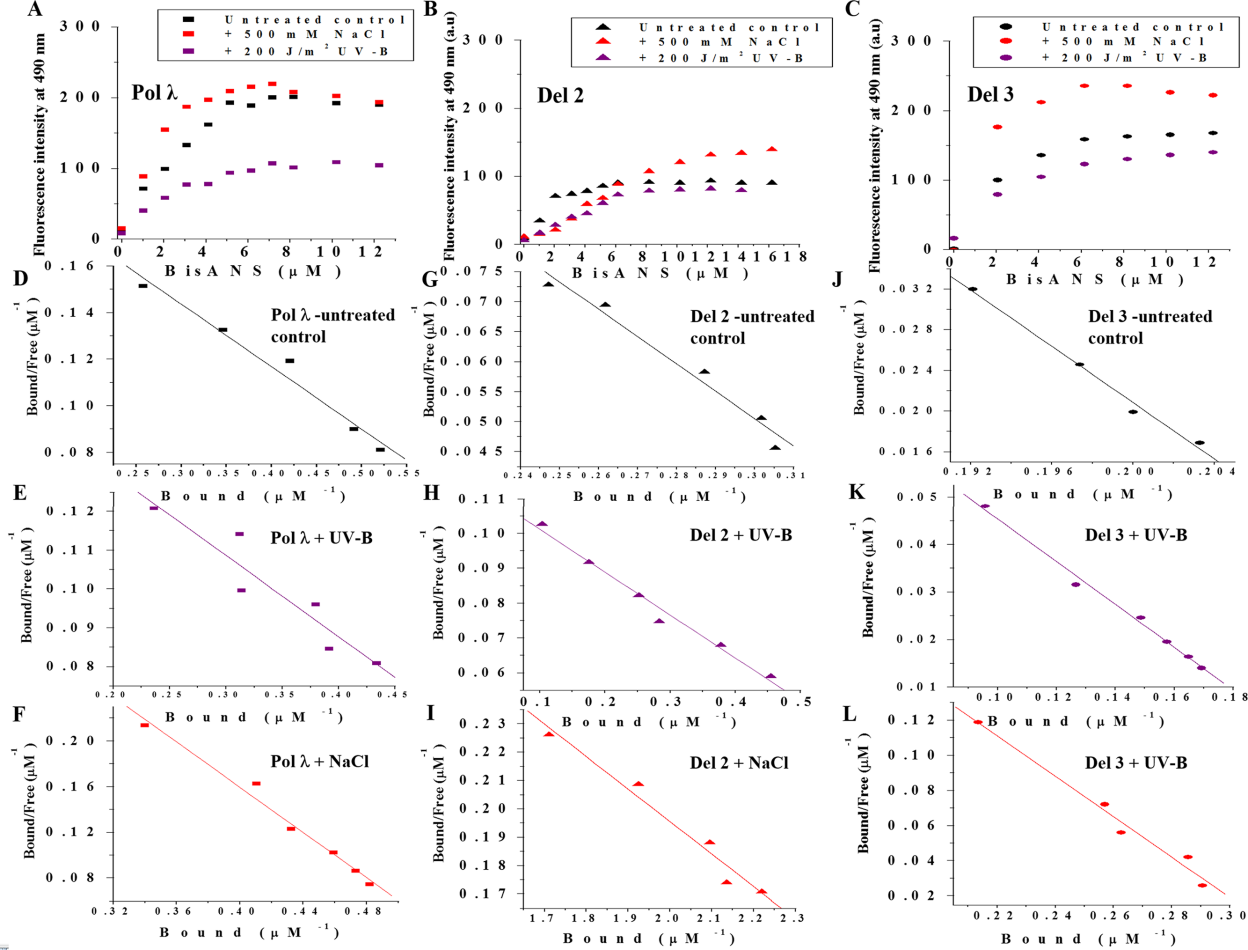
Changes in surface hydrophobicity of purified recombinant full length and N terminus deletion mutants of Pol λ following UV-B irradiation or high salt treatment. (A) Bis-ANS binding titration of untreated control, UV-B irradiated and high NaCl treated recombinant Pol λ. (B) Bis-ANS binding titration of untreated control, UV-B irradiated and high NaCl treated Del 2 protein. (C) Bis-ANS binding titration of untreated control, UV-B irradiated and high NaCl treated Del 3 protein. (D-F) Representative Scatchard plots of untreated control, UV-B irradiated and high NaCl treated recombinant Pol λ, (G-I) Del 2 protein and (J-L) Del 3 protein for the determination of stoichiometry (*n*) and the dissociation constant (k_d_) respectively. 0.02 mg/mL of each purified protein in 50 mM Tris-HCl buffer, pH 7.5 (containing 1 mM PMSF and 1 mM β-mercaptoethanol) was titrated by addition of aqueous solution of Bis-ANS. The excitation and emission wavelengths were 390 and 490 nm, respectively. The intensities at 490 nm, from the titration, were plotted as a function of Bis-ANS concentration. UV-B and NaCl treatment of purified recombinant proteins were carried out as described under ‘Materials and Methods’.

Surface hydrophobicity properties of proteins provide essential insight towards understanding the behavior of proteins under stressed conditions and potential interactions among protein partners [36]. Bis-ANS (4,4’-dianilino-1,1’-binaphthyl-5,5- disulfonic acid) is a conformation-sensitive hydrophobic probe with low fluorescence quantum yield in aqueous solution while becomes highly fluorescent upon binding to hydrophobic pockets [37]. Bis-ANS has been extensively used for probing the surface-exposed hydrophobicity of proteins in plethora of studies [29, 38–39]. We performed Bis-ANS binding assay to investigate the exposure of hydrophobic pockets on the surface of *Arabidopsis* Pol λ and its N-terminal deletion mutants following UV-B exposure or high salt treatment. For the quantification of the number of Bis-ANS binding sites (*n*) and the binding constant (*k*_*d*_), fluorometric titration of control and UV-B or NaCl treated proteins by Bis-ANS were carried out. The Bis-ANS titration curves for the protein samples have been shown in Fig 4A–4C, respectively. The data were analysed by plotting *v* versus *v*/*S* following the Scatchard equation: *v* = *n*—*k*_*d*_ [*v*/*S*], considering *n* number of identical non-interacting sites per subunit of the protein samples, where *v* and *S* represent the concentrations of bound and free Bis-ANS (μM), respectively, while *k*_*d*_ stands for the dissociation constant.

In addition, a reverse titration of Bis-ANS by protein samples were also carried out for the conversion of the change in fluorescence intensity into bound Bis-ANS [30]. The Scatchard plots, obtained from the reverse titration assay for the Bis-ANS-Pol λ, -Del 2 and-Del 3 binding under control or after UV-B or salt treatment are shown in Fig 4D–4F, 4G–4I and 4J–4L, respectively. The number of binding sites (*n*) and the dissociation constant for binding (*k*_*d*_) were calculated from the slope and intercepts of the plot and are summarized in Table 2.

**Table 2:** Determination of stoichiometry (*n*) and dissociation constant (K_d_) of recombinant full length and N terminus truncated forms of AtPolλ under control and after UV-B irradiation or high NaCl treatment.

System	*n*	Kd μM
Pol λ-untreated control	0.22 ± 0.01	3.72 ± 0.03
Pol λ + 200 J/m^2^ UV-B	0.17 ± 0.01	4.76 ± 0.04
Pol λ + 500 mM NaCl	0.55 ± 0.03	1.01 ± 0.07
Pol λ-Del 2-untreated control	0.18 ± 0.01	2.19 ± 0.05
Pol λ-Del 2 + 200 J/m^2^ UV-B	0.11 ± 0.02	8.14 ± 0.02
Pol λ-Del 2 + 500 mM NaCl	0.42 ± 0.04	8.73 ± 0.01
Pol λ-Del 3-untreated control	0.29 ± 0.02	0.72 ± 0.09
Pol λ-Del 3 + 200 J/m^2^ UV-B	0.1 ± 0.02	2.22 ± 0.02
Pol λ-Del 3 + 500 mM NaCl	0.37 ± 0.02	0.87 ± 0.09

# (Errors represent SD from triplicate measurements)

Comparison among untreated control protein samples have revealed that the hydrophobic sites decreased from full length Pol λ to Del 2, while increased slightly from Pol λ to Del 3 (Table 2), suggesting that whereas the deletion of BRCT domain has only marginal effect on surface hydrophobicity, removal of S-P region probably causes exposure of some hydrophobic groups to the surface of the globular structure of the protein. However, for full length Pol λ, after UV-B exposure, the hydrophobic sites (*n*) were slightly decreased from 0.22 to 0.17 with the concomitant increase in dissociation constant (*k*_*d*_) from 3.7 to 4.7 μM (Table 2). On the other hand, after 500 mM NaCl treatment, *n* for Pol λ increased ~2-fold with a decrease in *k*_*d*_ value by a factor of over 3 (Fig 4A, Table 2). The removal of N-terminal signal sequence (Del 1) did not cause any significant difference in the surface hydrophobicity properties under control or following stress treatment (data not shown). However, Del 2 protein showed a marginal decrease in *n* after UV-B exposure indicating loss of hydrophobic pockets possibly due to partial unfolding of the protein. This result was consistent with the tryptophan fluorescence spectra of Del 2 after UV-B exposure (Fig 4B, Table 2). Del 3 showed decrease in *n* after UV-B exposure (Fig 4C, Table 2). Interestingly, at high salinity condition, Pol λ and all of its N-terminally deletion mutants showed increase in *n* value. Since ionic interactions are suppressed with the increase in ionic strength of the medium, at higher NaCl concentrations hydrophobic interactions play major role and might result in the higher proportion of binding of the Bis-ANS molecule to the hydrophobic patches of the proteins.

### Thermodynamic stability of Pol λ and its N-terminal deletion mutants under genotoxic stress

**Fig 5:**
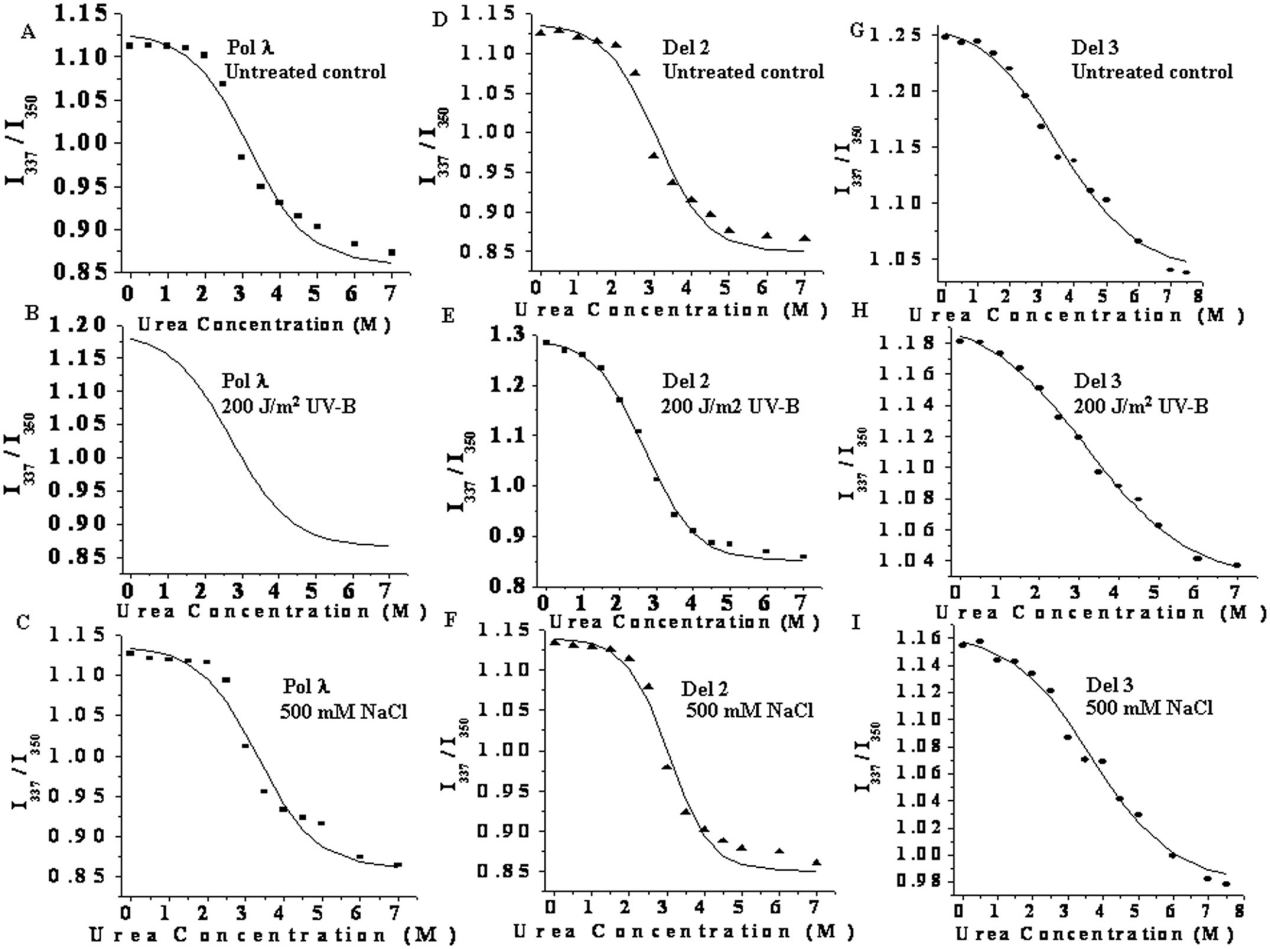
Thermodynamic stability of recombinant full length and the N terminus truncated forms of Pol λ under UV-B and high salt stress. (A-C) Urea induced denaturation profiles of recombinant full length Pol λ without treatment (A), after UV-B irradiation (B) or high salt treatment (C), respectively. (D-F) Urea induced denaturation profile of Del 2 protein without treatment (D), after UV-B irradiation (E), and high salt treatment (F). (G-I) Urea induced denaturation profile of DNA Pol λ-Del 3 protein without treatment (G) or after UV-B irradiation (H) and high salt treatment (I). The experiments were performed using 0.05 mg/mL of purified protein in 50 mM Tris-HCl buffer, pH 7.5 (containing 1 mM β-ME and 1 mM PMSF). Untreated control, UV-B irradiated (~200 J/m^2^ UV-B irradiation for 4 h at 25°C) and high salt (500 mM NaCl treatment for 2 h at 25°C) treated protein samples were incubated in presence of increasing concentrations of urea (0–8 mM) in total 600 μL of reaction volume for about 16 h at room temperature (25°C). Samples were excited at 295 nm and emission was scanned between 300 to 400 nm. Ratio of fluorescence intensity at 337 nm to the same at 350 nm was plotted as function of urea concentration.

To understand the thermodynamic basis of the stability of Pol λ under UV-B and NaCl stress and the relative sensitivity of the N-terminal deletion versions of the protein towards such stress conditions, we next studied the relative resistance of these proteins to urea-induced unfolding by measuring the tryptophan fluorescence of the protein solutions as a function of urea concentration in the absence or presence of stress treatment. Since the emission maxima shifted from 337 to over 350 nm with the increase in urea concentration from 0 to 8 M, the data were plotted as fluorescence intensity ratio (I337/I350) against urea concentration as described under ‘Materials and Methods’. Fig 5 shows the urea denaturation profiles of Pol λ, Del 2 and Del 3 proteins under control and after UV-B or NaCl treatment, respectively. The urea induced denaturation profiles yielded sigmoidal shape of the curves, indicating cooperativity. From the sigmoidal data analysis, the urea concentration, which caused 50% denaturation (C_0.5_) of the proteins, was obtained. For full length recombinant Pol λ, the C_0.5_ in the absence and presence of UV-B or salt stress were found to 2.98 (control), 2.71(UV-B) and 3.18 M urea, respectively. In case of Del 2 fragment of Pol λ, C_0.5_ under control condition was 2.93 M urea, while UV-B irradiated or salt treated Del 2 showed C_0.5_ values of 2.64 and 2.92 M urea, respectively. The C_0.5_ of Del 3 fragment (catalytic core PolX domain) changed from 3.66 (control) to 3.18 M urea after UV-B irradiation and to 3.81 M urea in presence of 500 mM NaCl (data not shown).

The standard free energy change (ΔG^0^) required to fully unfold the proteins from their native state in absence of urea was calculated by fitting the experimental denaturation profile according to a three-state model [31], assuming one intermediate and the thermodynamic parameters thus determined are summarized in Table 3. For recombinant full length Pol λ and its N-terminal deletion versions such as Del 2 and Del 3, the ΔG^0^_I_ were found to be higher than ΔG^0^_II_ both in the absence or presence of stress, suggesting that the intermediate is located away from the native state of the respective proteins in the free energy coordinate. However, under control condition, the stability (ΔG^0^) of Pol λ was different from Del 3 protein while ΔG^0^ of Del 2 did not differ significantly from Pol λ (Table 3). In the absence of UV-B or added NaCl (untreated control) ΔG^0^ of Del 3 (24.2 kJ/mole) was always higher than Pol λ (ΔG^0^: 19.7 kJ/mole) and Del 2 (18.2 kJ/mole), respectively. After UV-B treatment, ΔG^0^ of Pol λ was decreased from 19.7 kJ/mole to 17.6 kJ/mole, whereas for Del 2 and Del 3, ΔG^0^ decreased from 18.2 kJ/mole to 16.1 kJ/mole and from 24.2 kJ/mole to 22.4 kJ/mole, respectively following UV-B exposure. However, salt treatment showed somewhat stabilizing effect for all the protein samples. The ΔG^0^ of Del 1 did not show any appreciable difference from Pol λ either in absence or presence of stress (data not shown).

**Table 3:** Parameters of equilibrium urea unfolding of recombinant full length and N terminus truncated forms of AtPol under control and after UV-B irradiation or high NaCl treatment.

System	ΔG_I_ (kJ/mol)	ΔG_II_ (kJ/mol)	ΔG (kJ/mol)
Pol λ (Control)	14.9 ± 2.5	4.8 ± 1.1	19.7 ± 3.6
Pol λ + 200 J/m^2^ UV-B	13.6 ± 2.1	4.0 ± 0.8	17.6 ± 2.9
Pol λ + 500 mM NaCl	16.6 ± 3.2	5.7 ± 1.3	22.3 ± 4.5
Del 2 (Control)	14.9 ± 2.7	3.3 ± 0.6	18.2 ± 3.3
Del 2 + 200 J/m^2^ UV-B	12.4 ± 2.3	5.1 ± 1.1	16.1 ± 3.4
Del 2 + 500 mM NaCl	16 ± 2.1	2.4 ± 0.6	18.4 ± 2.7
Del 3 (Control)	15.8 ± 3.2	8.4 ± 2.1	24.2 ± 5.3
Del 3 + 200 J/m^2^ UV-B	11.8 ± 2.3	6.0 ± 1.1	22.4 ± 3.4
Del 3 + 500 mM NaCl	16.7 ± 3.4	8.6 ± 1.9	25.3 ± 5.3

# (Errors represent SD from triplicate measurements)

### Study of the secondary structure of recombinant Pol λ by circular dichroism and fourier transform infrared spectroscopy following UV-B and high salinity stress

**Fig 6:**
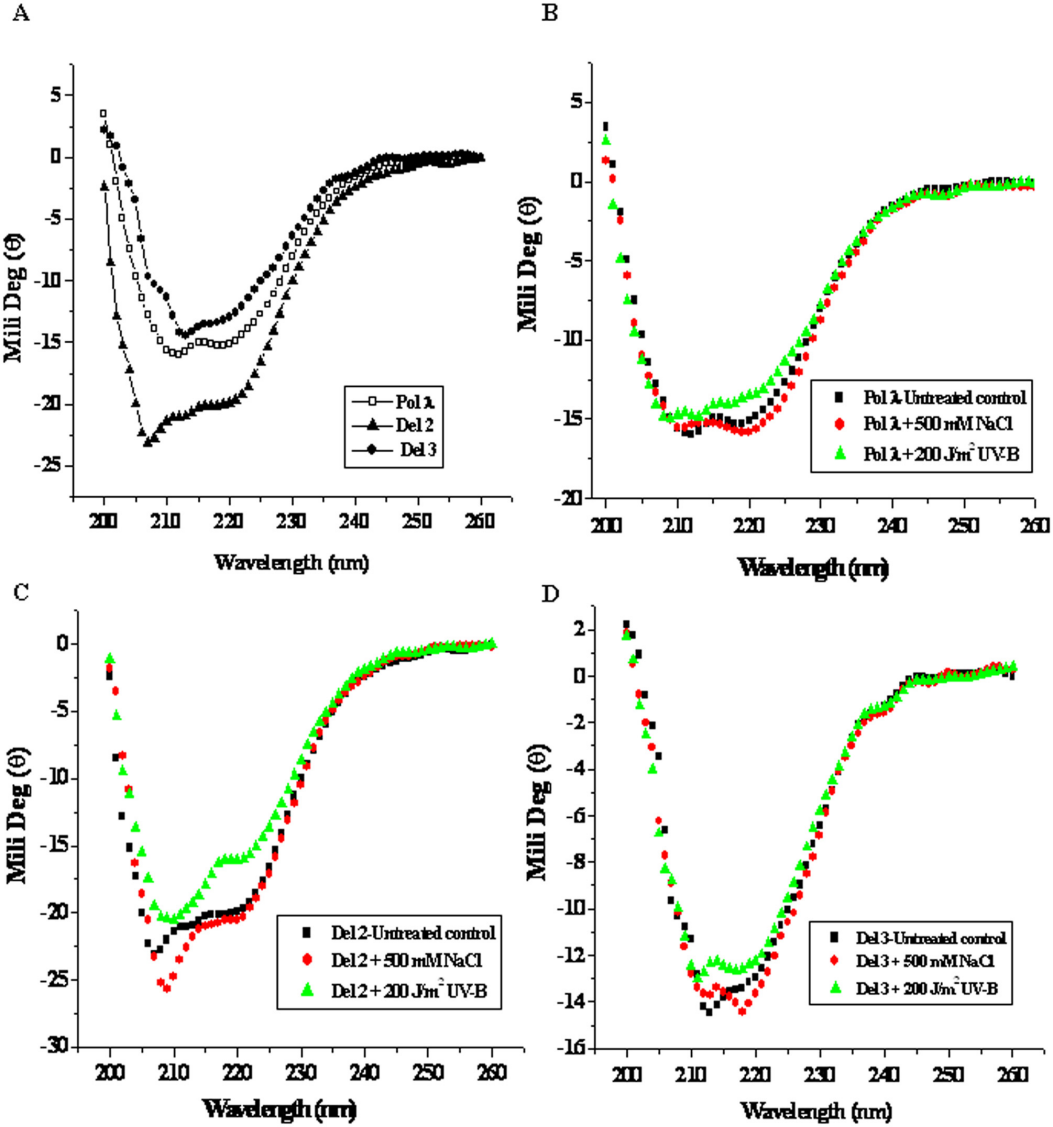
Analysis of changes in the secondary structure of full length Pol λ and it N-terminal deletion mutant proteins by far-UV CD spectroscopy. ~0.5 mg/ml purified recombinant protein samples in a total 1.5 mL reaction volume were UV-B irradiated with a dose of ~200 J/m^2^ for 4 h at 25°C or pre-incubated in presence of 500 mM NaCl for 2 h at 25°C. The far-UV CD spectra were recorded at 25°C in the wavelength range of 200–260 nm in a JASCO-810 Spectropolarimeter. (A) The far-UV CD spectra of untreated control Pol λ, Del 2 and Del 3 proteins. (B) The far-UV CD spectra of untreated control, UV-B and salt treated recombinant Pol λ. (C) The far-UV CD spectra of untreated control, UV-B and salt treated Del 2 protein. (D) The far-UV CD spectra of untreated control, UV-B and salt treated Del 3 protein.

We next analyzed the conformational change of Pol λ and the relative effects of the N-terminal domains on the structural stability of Pol λ at the level of secondary structure of the protein in the absence or presence of UV-B or NaCl stress. Far-UV circular dichroism (CD) spectra of recombinant Pol λ and its N-terminal deletion mutants (Del 2 and Del 3) were analyzed under control condition (without stress treatment) and after UV-B exposure or NaCl treatment of the protein samples as described under ‘Materials and Methods’. CD spectra of untreated control Pol λ and its N-terminal deletion versions are shown in Fig 6A, which indicated typical characteristics of alpha helical protein. However, control Del 2 protein showed appreciable difference from Pol λ and Del 3. (Fig 6A). On the other hand, whereas, CD spectral pattern of Pol λ and Del 3 remained mostly unaltered after UV-B exposure or salt treatment (Fig 6B and 6D), as compared with the untreated control condition, UV-B exposed Del 2 protein showed appreciable change in the CD spectrum (from w shape to v shape), suggesting some loss of secondary structural component. Salt treatment did not significantly alter the CD spectrum of Del 2 protein (Fig 6C).

In order to obtain quantitatively analyzed view of the CD spectral data, we fitted each spectrum with various secondary structure analysis programs such as, SELCON3, CONTIN, CDSSTR, CDNN, etc. [40]. Although the general agreement between the fitted parameters from different programs were not consistent and satisfactory, considerable increase in β-sheet structure at the expense of helical structure and increase in random coil contribution did not occur as a result of removal of the N-terminal domains of Pol λ or following exposure to UV-B or high NaCl (Table 4). Pol λ and its N-terminal mutants showed α-helix in the range of 30–35%. The variations in most cases were within the limits of experimental error. On the other hand, β-sheet, unordered content and turn were found in the range of ~16–20%, 20% and 32%, respectively. After UV-B and salt stress, Pol λ showed decreased α-helix content with some increase in the unordered structure. Similar pattern was observed with Del 2, however the extent of loss of α-helix content in Del 2 after UV-B exposure was ~22% as compared with Pol λ where ~14% loss of α-helix content was obtained. The loss of α-helix was complemented with some increase in β-sheet and random coil structure.

**Table 4:** Analysis of changes in the secondary structure compositions (in percentage) of full length and the N terminus truncated forms of recombinant purified Pol λ after UV-B and high salt stress by far-UV CD spectroscopy.

System	α-Helix (%)	β-Sheet (%)	Unordered (%)	Turn (%)
Pol λ Control	35 ± 3	17 ± 2	28 ± 3	20 ± 2
Pol λ+ 200 J/m^2^ UV-B	30 ± 4	18 ± 2	33 ± 3	20 ± 2
Pol λ+ 500 mM NaCl	32 ± 2	16± 1	31 ± 2	21 ± 3
Pol λ-Del2 Control	38 ± 3	12 ± 1	29 ± 4	21 ± 3
Pol λ-Del2+ 200 J/m^2^ UV-B	30 ± 3	16 ± 3	33 ± 4	21 ± 2
Pol λ-Del2+ 500 mM NaCl	36 ± 4	10 ± 2	32 ± 3	22 ± 2
Pol λ-Del3 Control	31 ± 3	20 ± 2	32 ± 3	17 ± 3
Pol λ-Del3+ 200 J/m^2^ UV-B	29 ± 3	19 ± 3	33 ± 2	19 ± 1
Pol λ-Del3+ 500 mM NaCl	33 ± 2	19 ± 2	32 ± 3	16 ± 1

# (Errors represent SD from triplicate measurements)

To further substantiate the secondary structure related information of the proteins obtained from the CD spectra, we next carried out the fourier transform infrared spectroscopy (FT-IR) using the purified recombinant protein samples. The amide I band of protein FT-IR showed overlapping bands from different secondary structural elements, which were resolved by Fourier self-deconvolution. The original amide I band and the curve fitting have been shown in S8 Fig. The percentage of individual secondary structural elements was obtained by using the GRAMS AI curve fitting program (S9 Fig). The percentage of secondary structures thus obtained has been shown in S1 Table. The percentage of secondary structure obtained from fitting the FTIR spectrum of the Pol λ and its N-terminal deletion mutants in presence and absence of stress in general was consistent with the CD data. Loss of α-helix content in Pol λ after UV-B exposure was 13%, while it was 20% in Del 2 protein under similar condition (S1 Table). The loss of the helical content in Del 2 seems mostly due to unfolding of the protein as indicated by the increased random coil structure of the protein. Del 3 did not show any appreciable change in its structure in presence and absence of stress (S9 Fig and S1 Table). *In vitro* activity assay using purified recombinant proteins have also indicated compromised DNA polymerase activity of 4 h UV-B irradiated Del 2 fragment of Pol λ, while UV-B did not drastically reduce the polymerase activity of the catalytic core PolX domain (Del 3), indicating the regulatory function of the S-P region (S10A–S10D Fig). Interestingly, considerable activity was detected with full length and N-terminal deletion forms of Pol λ in presence of up to 400–500 mM NaCl, (S10E–S10H Fig), indicating maintenance of active conformation of the protein in presence of high salt. Furthermore, as expected, the major part of the DNA polymerase activity has been found to be associated with C terminus catalytic core PolX domain (Del 3) of the protein, while the N terminus S-P region suppressed Pol λ activity (S10A, S10D, S10E and S10H Fig, 0 h time points). These results were consistent with human DNA Pol λ, where the N terminus Ser-Pro rich domain has been shown to exert similar inhibitory function on the DNA polymerase activity of the enzyme [41].

### Pol λ does not form aggregates under UV-B and high salinity stress

**Fig 7:**
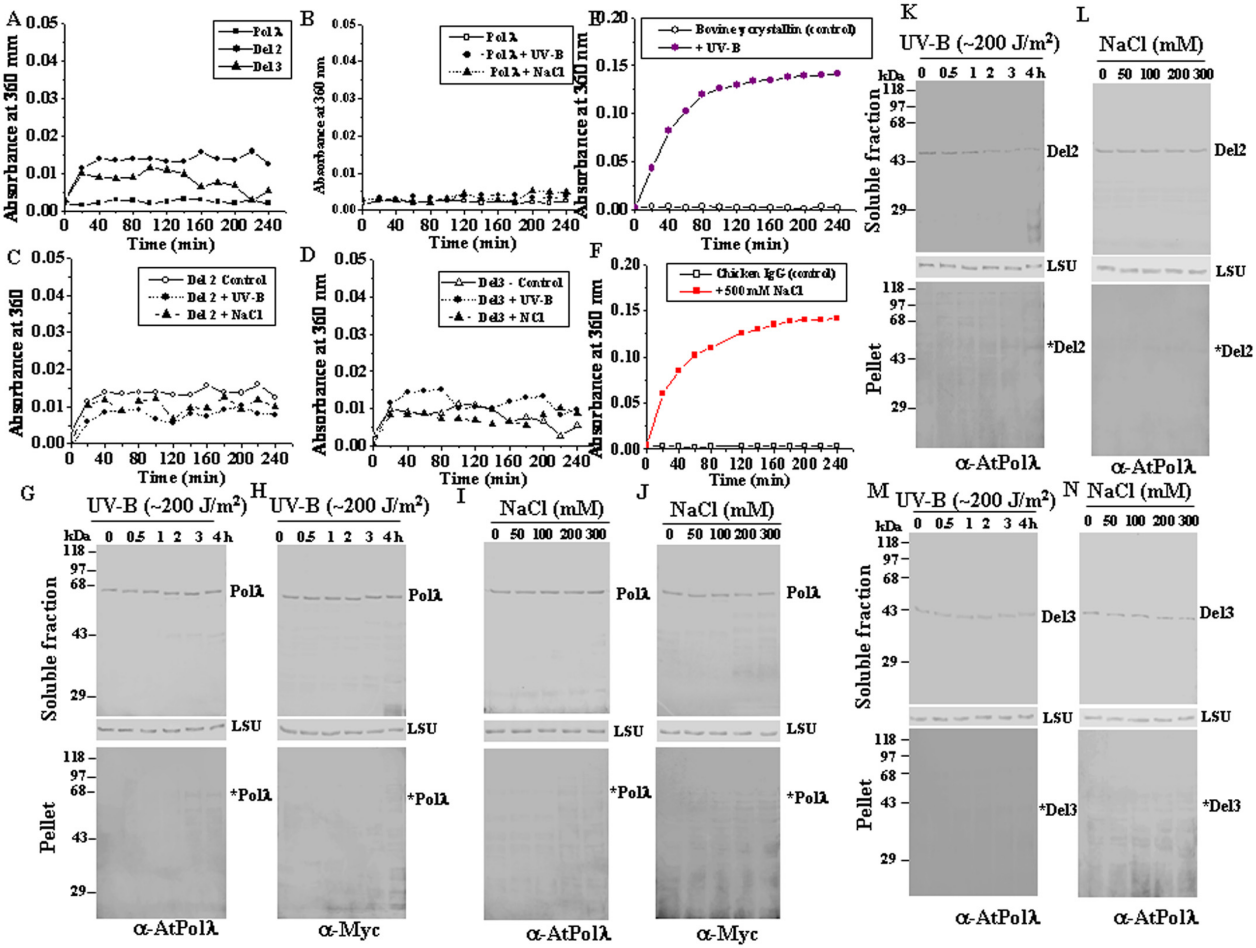
Analysis of the possibility of UV-B and salt-induced aggregation of Pol λ *in vitro* and *in vivo*. A) Aggregation assay using untreated control purified recombinant full length and N terminus deletion mutants of Pol λ. Aggregation assay using (B) untreated control, UV-B irradiated and salt treated purified recombinant full length, (C) Del 2 and (D) Del 3 proteins, respectively. (E) UV-B mediated aggregation of purified bovine γ crystalline. (F) Aggregation of high salt treated chicken IgG. 0.1 mg/ml of purified untreated control, UV-B irradiated (~200 J/m^2^ of UV-B treatment for 4 h at 25°C) or salt treated (500 mM NaCl treatment for 2 h at 25°C) protein samples in 50 mM Tris-HCl, pH 7.5 (with 1 mM PMSF and 1 mM β-mercaptothanol) were subjected to static light scattering at 360 nm. Absorbance was measured over a time course of 4 h at room temperature (25°C). Influence of UV-B irradiation and high salinity stress on Aggregation of Pol λ and its N terminus truncated forms *in vivo*. (G) and (H) Immunoblotting of protein extracts from soluble and insoluble (pellet) fractions of UV-B irradiated protoplasts from wild-type *Arabidopsis* (G) and tobacco leaf mesophyll cells transiently expressing AtPolλ (H). (I) and (J) Immunoblotting of protein extracts from soluble and insoluble (pellet) fractions from 7-days-old wild-type *Arabidopsis* seedlings (I) and *Agro*-infiltrated tobacco leaves (transiently expressing AtPolλ) exposed to increasing concentrations of NaCl (J). (K) and (L) Immunoblotting of protein extract from soluble and insoluble (pellet) fractions from UV-B irradiated protoplasts of transgenic *atpolλ-1* seedlings expressing Pol λ-Del-2 protein (*atpolλ-1* ^*Pol λ-Del 2*^) (K) or 7-days-old *atpolλ-1* ^*Pol λ-Del 2*^ seedlings exposed to increasing concentrations of NaCl (L). (M) and (N) Immunoblotting of protein extracts from soluble and insoluble (pellet) fractions from UV-B irradiated protoplasts of transgenic *atpolλ-1* seedlings expressing Pol λ-Del 3 protein (*atpolλ-1* ^*Pol λ-Del 3*^) (M) or 7-days-old *atpolλ-1* ^*Pol λ-Del 2*^ seedlings exposed to increasing concentrations of NaCl (N). Positions of DNA Pol λ in the soluble and pellet fractions have been indicated. Size markers are shown on the left of gel images. Ponceau S stained membranes with the soluble protein extracts showing RuBisCo large subunit (LSU) have been shown as loading controls (middle panel, Fig G-N). Representative data in Fig G-N are from three trials with similar results. Immunoblotting of protein extracts from *Arabidopsis* (Col-0 and transgenic lines) protoplasts or seedlings were carried out using affinity purified anti-AtPolλ polyclonal antibody (1:250 dilutions) while anti-c-Myc antibody (1:1000 dilutions) was used for immunodetection of DNA Pol λ in protein extracts from tobacco leaves or mesophyll cell protoplasts ectopically expressing AtPolλ. *atpolλ-1* is a null mutant line, devoid of AtPolλ expression. Representative gel images from at least three independent trials are shown.

UV-B light frequently causes protein misfolding and aggregation by modification or destruction of amino acid residues [42], while high salinity also acts as a major genotoxic stress for plants, affecting protein unfolding and solubility. Based on this, we next studied whether exposure to UV-B or high NaCl concentrations affect the stability of Pol λ *in vitro* and *in vivo*. Light scattering experiments were carried out using purified recombinant protein samples to monitor aggregation after UV-B exposure or high NaCl treatment. The Del 2 and Del 3 fragments of Pol λ showed slightly higher light scattering at room temperature (25°C) than full length Pol λ (Fig 7A). Similar pattern was also noted at 37°C (data not shown). However, no significant increase in light scattering could be detected after UV-B irradiation or high NaCl treatment either at 25°C (Fig 7B–7D) or at 37°C (data not shown). The light scattering behaviour of Del 1 in the absence or presence of stress was similar to recombinant full length Pol λ (data not shown). UV-B induced aggregation of purified bovine γ-crystallin and aggregation of chicken IgG in presence of 500 mM NaCl were used as positive controls (Fig 7E and 7F) to validate the experimental conditions. Taken together, these results indicate that ~200 J/m^2^ of UV-B irradiation for up to 4 h or high salinity (500 mM NaCl) did not cause any significant level of aggregation of Pol λ or its N-terminal deletion mutants *in vitro*.

**Fig 8:**
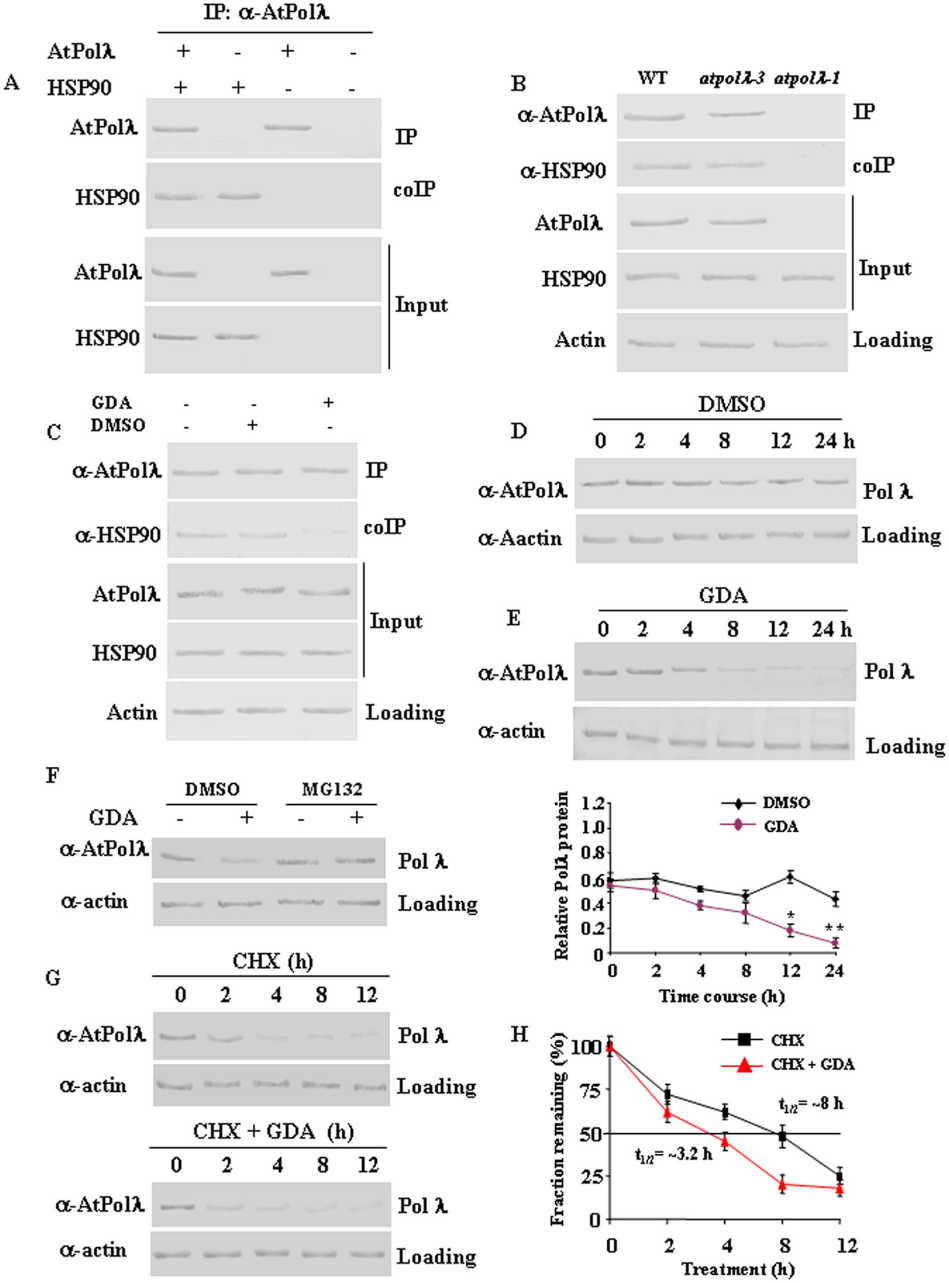
Pol λ interacts with HSP90 *in vivo*. (A) Leaves from *Agro*-infiltrated tobacco (*N*. *benthamiana*) plants, transiently and ectopically expressing AtPolλ-C-TAP (DKLAT1G10520.1) and AtHSP90.1-C-TAP (DKLAT5G52640) were harvested and cross-linked with 1% formaldehyde. Protein extracts from the harvested tobacco leaves was immunoprecipitated with affinity purified anti-AtPolλ polyclonal antibody and anti-AtPolλ immunoprecipitate (IP) was probed with anti-HSP90 antibody (1:500 dilution) (coIP). (B) Pol λ and HSP90 interacts in *Arabidopsis* at endogenous levels. *Arabidopsis* seedlings were grown for 7–10 under long day cycles, seedlings were harvested, cross-linked with 1% formaldehyde and protein extract was prepared. The protein extract was immunoprecipitated with affinity purified anti-AtPolλ polyclonal antibody and immune complexes from protein extracts were probed for endogenous AtPolλ (IP) and coimmunopreciptated endogenous HSP90 (coIP) using affinity purified anti-AtPolλ polyclonal antibody (1:250 dilution) and anti-HSP90 antibody (1:500 dilution), respectively. Actin, detected by anti-actin monoclonal antibody was used as loading control. Protein extracts from *atpolλ-3* was used as positive control while *atpolλ-1* was used as negative control. *atpolλ-1* is a null mutant line, devoid of Pol λ expression and *atpolλ-3* is Pol λ protein positive mutant allele. Migration positions of Pol λ or HSP90 have been indicated. (C) Geldanamycin (GDA) treatment inhibits Pol λ-HSP90 interaction. 7-days-old *Arabidopsis* seedlings (Col-0) were treated with 5 μM GDA or vehicle (1% DMSO) for 8 h, protein extracts prepared and immunoprecipitated with anti-AtPolλ polyclonal antibody. Immune complexes from protein extracts were probed for endogenous AtPolλ (IP) and coimmunopreciptated endogenous HSP90 (coIP). Actin was used as loading control. (D) and (E) Geldanamycin treatment reduces Pol λ protein level. 7-days-old *Arabidopsis* seedlings (Col-0) were treated with 1% DMSO (D) or 5 μM GDA (E) for the indicated time points and protein extracts were immunoblotted as indicated. Actin was used as loading control. Quantifications of the data in (D) and (E) (lower graph). *P <0.05, *P <0.01 relative to respective controls (*n* = 3). (F) 7-days-old wild-type *Arabidopsis* seedlings were treated with 5 μM GDA for 8 h with vehicle (DMSO) or with 50 μM MG132 for at least 4 h. Protein extracts were then immunoblotted using anti-AtPolλ polyclonal antibody. Actin was used as loading control. (G) 7-days-old wild-type *Arabidopsis* seedlings were treated with 200 μM cycloheximide in the absence (upper panel) or presence of 5 μM GDA for the indicated time points (lower panel) and protein extracts were immunoblotted as indicated. (H) Pol λ signals were quantified and normalized against actin signals. Data shown are means ± SD of three independent replications (lower graph). Representative gel images from at least three independent trials are shown.

We next looked into the possibility of aggregation of Pol λ *in vivo* in the absence or presence of UV-B or high salt stress. The extent of aggregation was determined from the loss of relative amount of target protein in the soluble fractions and recovery in the insoluble fractions as measured by immunoblot analysis. Intact protoplasts from wild-type *Arabidopsis* (expressing the endogenous *Pol λ*) and tobacco (*Nicotiana benthamiana*) leaf mesophyll cell (transiently expressing C-terminal TAP-tagged *Arabidopsis Pol λ* under CaMV-35S promoter) were exposed to UV-B light (~200 J/m^2^) or 7-days-old wild-type *Arabidopsis* seedlings or *Agro*-infiltrated tobacco leaves (transiently expressing *Pol λ*) were subjected to increasing concentrations of NaCl (0–300 mM) treatment (S1 File). Immunoblotting of soluble and insoluble protein extracts from UV-B and high salt treated seedling samples have detected localization of Pol λ mostly in the soluble fractions (Fig 8G–8J, upper panels). Only marginal loss of Pol λ band intensity, if any, was detected in the soluble fraction in both *Arabidopsis* and tobacco under UV-B exposure and salinity stress (Fig 7G–7J, upper and lower panels, respectively).

To analyse the effects of deletion of N-terminal domains on the aggregation of Pol λ in presence of UV-B or salinity stress *in vivo*, immunoblotting was carried out using protein extracts from control and UV-B irradiated leaf mesophyll cell protoplasts from stable transgenic *atpolλ-1* mutant plants (devoid of *AtPolλ* expression) expressing *Pol λ*: *Del2* and *Del3* cDNA fragments under the control of the CaMV-35S constitutive promoter (*atpolλ-1* ^*Pol λ-Del2*^ and *atpolλ-1* ^*Pol λ-Del3*^) (S11 Fig). For salt stress, 7-days-old transgenic *atpolλ-1* ^*Pol λ-Del2*^ and *atpolλ-1* ^*Pol λ-Del3*^ seedlings were exposed to increasing concentrations of NaCl at room temperature and then protein extracts were prepared. After UV-B or salinity stress, as like Pol λ, major amounts of Del 2 and Del 3 proteins were mainly found in the soluble fractions, however, detectable levels of both Del 2 and Del 3 proteins were also observed in the insoluble fractions following stress treatment (Fig 7K–7N, upper and lower panels). UV-B irradiated protoplasts expressing Del 2 showed a progressive loss of Del 2 band (~46-kDa) intensity in the soluble protein fraction along with the gradual accumulation of Del 2 in pellet fraction with increasing duration of exposure to UV-B light (Fig 7K). In addition, the relative level of Del 3 protein in the soluble fraction was also decreased in presence of 200–300 mM NaCl with the concomitant appearance of detectable level of Del 3 protein in the pellet fraction under identical conditions (Fig 7N). Taken together, these results may indicate partial unfolding of Del 2 fragment of Pol λ particularly under UV-B stress and also some extent of high salinity mediated unfolding of Del 3, resulting in the availability of the proteins in the insoluble fraction. The possibility of protein aggregation cannot be completely ruled out. However, light scattering experiments did not indicate any significant level of protein aggregation after stress treatment.

### Pol λ is a client of the eukaryotic molecular chaperone HSP90

In mammals, the essential molecular chaperone HSP90 interacts directly with many important components of DNA damage response pathway to regulate the stability and accumulation of DNA damage repair proteins [43–44]. Based on this, we next tested whether Pol λ interacts with HSP90 in plant cell to understand the regulation of stability of this protein at the molecular level. To study the possible interaction *in vivo*, we carried out coimmunoprecipitation experiments (Co-IPs) from transiently coexpressed *Arabidopsis* DNA Pol λ (AtPolλ) and HSP90 (both as C-terminal TAP tag constructs) in *Nicotiana benthamiana* leaves. HSP90 was successfully detected in AtPolλ immunoprecipitates from tobacco leaf tissues ectopically expressing AtPolλ-TAP and AtHSP90.1-TAP under the CaMV-35S promoter, indicating that a Pol λ-HSP90 complex can form *in vivo* (Fig 8A). Immunoprecipitations performed using an anti-AtPolλ antibody on extracts from wild-type *Arabidopsis* seedlings detected HSP90 in the immunoprecipitates (Fig 8B). HSP90 was not coimmunoprecipitated from *atpolλ-1* null background, while HSP90 was detected in coimmunoprecipitates using protein extracts from *atpolλ-3* background, the protein-positive allele *AtPolλ* (Fig 8B). In addition, the interaction was strongly diminished in presence of geldanamycin (Fig 8C), a specific inhibitor of HSP90 [45], suggesting that HSP90 specifically interacts with Pol λ. Together, these results indicate that Pol λ and HSP90 interact *in vivo* at endogenous levels and support the idea that Pol λ is a client of HSP90.

### Geldanamycin treatment probably induces proteasomal degradation of Pol λ

To further understand the functional significance of the interaction between HSP90 and Pol λ, we next examined the effect of geldanamycin, which acts as a specific inhibitor of HSP90, on Pol λ protein levels in *Arabidopsis* seedlings. 7-days-old wild-type *Arabidopsis* seedlings were treated with 5 μM of geldanamycin (GDA) or vehicle (DMSO) for the indicated time points (Fig 8D and 8E) and Pol λ protein levels were analysed by immunoblotting. After DMSO treatment, the normal oscillation in Pol λ protein level was observed (Fig 8D), whereas GDA treatment caused significant reduction in Pol λ levels (Fig 8E, lower panel graph).

Geldanamycin is known to induce proteasomal degradation of HSP90 clients [46]. Therefore, we next examined effects of proteasomal inhibitors. As shown in Fig 8F, MG-132 treatment of wild-type *Arabidopsis* seedlings appreciably compromised the geldanamycin mediated reduction of Pol λ protein levels. Treatment of *Arabidopsis* seedlings with translation inhibitor cycloheximide indicated that geldanamycin treatment considerably reduced the half-life of Pol λ (Fig 8G and 8H). On the other hand, geldanamycin treatment showed little effect on Pol λ mRNA levels in *Arabidopsis* seedlings (S12 Fig). Together, these results indicate that geldanamycin induced depletion in HSP90 is most likely associated with the accelerated proteasomal degradation of Pol λ in *Arabidopsis* and thus link the possibility of HSP90 mediated regulation of stability of Pol λ.

## Discussion

Earlier, we have reported that UV-B exposure and high salinity treatment leads to enhanced accumulation of Pol λ protein in *Arabidopsis* seedlings. Overexpression of *Pol λ* cDNA in wild-type *Arabidopsis* notably decreased the UV-B and high salinity mediated growth inhibition of seedlings as compared to non-overexpressor wild-type plants and *Pol λ* loss-of-function mutants. In addition, Pol λ overexpression has increased the efficiency of repair of UV-B and high NaCl induced DNA damage in the transgenic wild-type *Arabidopsis* and *atpolλ* mutant seedlings. Pol λ overproduction was also found to confer salt tolerance effect in the model prokaryotic system *E*. *coli* and unicellular eukaryote yeast (*Saccharomyces cerevisiae*) [19, 21]. These results have highlighted that a stress tolerance trait is associated with Pol λ function. Therefore, Pol λ overexpression probably facilitate to enhance the repair efficiency of DNA damages generated by UV-B and high salinity and confers a better growth response under such stress conditions. In mammalian system, considerable research has been carried out to get structural insight into the biological functions of Pol λ [9]. However, corresponding information on the structure-function properties of this important component of plant nuclear DNA damage repair machinery in connection with the physical and molecular basis of stability of this protein under abiotic stress condition is largely limited. In this study, we have investigated the physical and molecular basis of stability of *Arabidopsis* Pol λ under UV-B and salt stress using biophysical and molecular approaches. Along with the purified recombinant full length protein, we have utilized the N-terminal deletion versions of Pol λ lacking the BRCT domain and Ser-Pro rich region to further understand the relative contributions of the N-terminal regions of Pol λ in regulating the stability of the C-terminal catalytic core PolX domain and the overall structure of the protein.

Progressive decline in tryptophan fluorescence intensity of Pol λ and the other N-terminal deletion forms (Del 1, Del 2 and Del 3, respectively) with the increasing duration of exposure to UV-B light (Fig 1A–1D) have suggested structural alteration of the proteins. Comparative analysis of normalized tryptophan fluorescence intensities of the WT Pol λ and its N-terminal deletion mutants have revealed higher contribution of one or both the tryptophan residues located within the BRCT domain (Del 2) to the overall fluorescence intensity compared to the other three tryptophan residues located within the catalytic core PolX domain (Del 3) (Fig 1E and S1B Fig).

Except the Del 2 fragment, for WT Pol λ and the other N-terminal deletion forms, the emission maximum remained unaltered after 4 h of UV-B exposure (200 J/m^2^), indicating that for these proteins, UV-B induced changes did not significantly affect the tryptophan microenvironment. In contrast, along with the decreased intensity, Del 2 fragment of Pol λ showed a clear shift of the emission maximum from 340 nm to 350 nm after 4 h of UV-B exposure, suggesting that UV-B light caused exposure of tryptophan residues located within the C-terminal catalytic core PolX domain of Del 2 protein (Fig 1F). The Del 3 fragment, which is derived from the deletion of S-P region of Del 2 fragment of Pol λ, did not display any shift in emission maximum, indicating that S-P region affects structural stability of the Del 2 fragment. The S-P region contains repeats of proline residues which are known structure modulator and impose structural constrains [47–48]. In the native protein (WT Pol λ), presence of other domains prevents structural alteration which could lead to tryptophan exposure. Nevertheless, the Del 2 fragment of Pol λ, which lacks the N-terminal 99 amino acid residues of Pol λ, appeared to be destabilized by the proline residues of the S-P region as its population density becomes higher in Del 2 compared to WT Pol λ. This notion was supported by the increase in thermodynamic stability of Del 3 fragment (following deletion of the S-P region from Del 2) as found in urea denaturation profiles (Table 3).

Following UV-B exposure, tryptophan residues frequently get oxidized to form *N*-formylkynurenine, which shows fluorescence maximum at 440 nm. UV-B exposure decreased tryptophan fluorescence intensities (Fig 1A–1D) with the concomitant increase in *N*- formylkynurenine emission (Fig 2B). Maximum increase in *N*-formylkynurenine was observed for UV-B irradiated Del 2, while as like full length Pol λ, tryptophan oxidation to *N*-formylkynurenine was least in Del 1. Together, these results support our idea that UV-B irradiation mainly exposes the tryptophan residues of the catalytic core PolX domain, resulting in enhanced sensitivity of Del 2 towards UV-B as compared with the other regions of Pol λ. Tryptophan fluorescence quenching experiments also showed similar indication as the K_SV_ value of Del 2 increased after UV-B irradiation. Earlier studies have indicated that proline prefers *cis*-peptide conformation over *trans*, preferred by most of the other amino acids in peptide bond formation. Increase in population density of proline in Del 2 may add this constrain to the peptide backbone to cause partial exposure of tryptophan residues, making it vulnerable towards UV-B radiation. On the other hand, the two tryptophan residues located within the BRCT domain seemed to remain largely protected from UV-B induced damage as observed from the tryptophan fluorescence spectra of Del 1 under UV-B stress. Furthermore, N-formylkynurenine production was ~5 to 6 fold higher in case of BSA than *Arabidopsis* Pol λ after 4 h of UV-B exposure (Fig 2E). This suggests that Pol λ has the ability to chaperone the tryptophan residues against UV-B damage compared to the control protein BSA.

Progressive decrease in tryptophan fluorescence intensity of recombinant Pol λ (WT) and the other N-terminal deletion fragments was also observed in presence of increasing NaCl concentrations (Fig 3). Although loss of intensity might indicate some structural alterations, the lack of shift of emission maximum of the proteins suggested that NaCl was unable to alter the polarity of tryptophan microenvironment as the fluorescence contributing tryptophan residues were not completely exposed to aqueous environment. Tryptophan residues remain buried within the globular structure of the protein and hence the salt mediated changes may not interrupt the tryptophan microenvironment. However, the decline in tryptophan fluorescence intensity after salt treatment probably result from the changes in the distribution of the neighbouring charge groups to help energy transfer [37]. It was interesting to note the enhanced sensitivity of Del 3 protein in presence of 200 mM NaCl (~35% drop of tryptophan fluorescence intensity) as compared to full length Pol λ, Del 1 and Del 2 proteins under similar condition (Fig 3A–3D). However, tryptophan fluorescence intensity in Del 3 did not significantly decrease further with increasing NaCl concentrations. WT Pol λ, Del 1 and Del 2 showed gradual decrease in tryptophan fluorescence with increasing NaCl concentrations. These results have ruled out the existence of any static quenching since the amount of quenching of intensity was not clearly proportional to NaCl concentrations for the proteins [35]. The static quenching generally arises due to binding of NaCl to the proteins and therefore, the differential decrease in tryptophan fluorescence intensity of the proteins in presence of increasing NaCl concentration may indicate changes in the ionic environment at the protein surface [35, 49].

The exposure or destruction of hydrophobic pockets on the surface of recombinant full length Pol λ and its N-terminal deletion forms in presence and absence UV-B and high salinity stress was analyzed by the highly sensitive Bis-ANS binding assay (Fig 4, Table 2). Since, Del 2 (control) was found to be partially unfolded due to the additional structural constrain imposed by proline repeats, showed lower hydrophobic clefts as compared to full length Pol λ and other deletion forms. Absence of proline repeats in Del 3 enhanced the structural stability with higher hydrophobic binding pockets. However, in general, WT Pol λ and its deletion mutants showed reduction in hydrophobic binding sites upon exposure to UV-B light. The apparent cause of this observation might be the formation of kynurenine and its derivatives due to oxidation of tryptophan residues. The additional carbonyl group in the kynurenine increases the polarity, thereby reduces the binding affinity of the protein to Bis-ANS [50]. This leakage in hydrophobic core may lead to loss of structural integrity of the protein. It was interesting to note the higher K_SV_ value of UV-B induced Del 2 also corroborates well with this notion. In case of salinity stress, we observed increase in number of Bis-ANS binding pockets for Pol λ and other deletion mutants (Table 2), indicating exposure of hydrophobic pockets, which probably favour hydrophobic interactions by reducing the ionic interactions under high salinity.

Urea denaturation profiles of the proteins (Fig 5, Table 3) indicated marginal effect on the stability of Pol λ after removal of the N-terminal BRCT domain as the ΔG^0^_Del2_ -ΔG^0^_Polλ_ was -1.46 kJ/mol (negative sign indicates destabilization). On the other hand, further removal of the S-P region from the Del 2 fragment resulted in appreciable gain of stability of the C-terminal catalytic core PolX domain (Del 3) with the ΔG^0^_Del3_-ΔG^0^_Del2_ was found to be 6.0 kJ/mol, suggesting the destabilizing effect of the S-P region of Pol λ. UV-B exposure for 4 h (~200 J/m^2^) destabilized the proteins and the effect was more pronounced in case of Del 2 where the ΔΔG^0^_Del2-UV-B_ value (ΔG^0^_UV-B—_ΔG^0^_control_) was -2.1 kJ/mol, while the ΔΔG^0^_Polλ-UV-B_ and ΔΔG^0^_Del3-UV-B_ values were found to be -2.06 and -1.8 kJ/mol, respectively under UV-B stress.

Recombinant full length Pol λ showed gain of stability in presence of 500 mM NaCl, as the ΔΔG^0^_Polλ-salt_ (ΔG^0^_salt—_ΔG^0^_control_) was 2.64 kJ/mol. Del 2, on the other hand, did not gain additional stability in presence of salt as the ΔΔG^0^_Del2-salt_ was only 0.2 kJ/mol. Marginal gain of stability was observed for Del 3 in presence of salt (ΔΔG^0^_Del3-salt_: 1.1 kJ/mol). We anticipate that there are three possible mechanisms, which may explain the apparent gain of stability of the protein in presence of added salt. Salts are known to stabilize protein conformation through selective binding [51–52]. Under neutral pH condition, Pol λ and its deletion mutants are negatively charged and specific binding of sodium ions may reduce the local negative charge, lowering the unfavorable electrostatic interaction and increasing the stability of the protein. However, non-linear quenching of tryptophan fluorescence with NaCl concentration speaks against the specific cation binding hypothesis as it indicates less or no binding of Na^+^ ions to the nearby regions of tryptophan. The second mechanism is the Debye screening, involving selection of repulsive electrostatic interactions in the native state of the proteins by the mobile ions, thereby reducing the free energy of the ground state. It may involve the screening of favorable electrostatic interactions in the unfolded state thereby increasing the free energy of the unfolded state. Another possible mechanism which explains the increase in stability of the proteins at high salinity involves the Hofmeister effect, which arises as many salts at higher concentration promote the hydrophobic interactions, increasing the stability of the proteins. The effect originates due to the ability of the salt to perturb protein structure and H-bonding properties of water [53]. Based on this, we anticipate that though Debye screening may contribute to Pol λ stability and its variants at higher NaCl concentrations, Hofmeister effect may be the primary mechanism for protein stability under high NaCl concentration.

UV-B exposure and salinity stress indicated some conformational changes in Pol λ and its N terminal deletion mutants. However, CD and FT-IR studies did not show appreciable change in the protein secondary structure either in presence of UV-B or salt stress, implying that the change mostly occurred at the tertiary level. It was also interesting to note considerable photo degradation of recombinant WT and the N-terminal deletion forms Pol λ at higher UV-B dose (312–546 J/m^2^), while NaCl concentrations beyond 800 mM caused protein precipitation (data not shown).

Protein stability refers to the maintenance of a defined functional conformation under extreme conditions and may involve all levels of the hierarchy of protein structure: local packing of the polypeptide chain, secondary and supersecondary structural elements, domains and subunits. UV-B light induces modification or destruction of amino acid residues, mainly via photooxidation of aromatic amino acids and thus may lead to inactivation of whole protein. On the other hand, High salinity stress causes protein damage by inducing protein unfolding and aggregation. Interestingly, the halophilic proteins, for example, require relatively higher salt concentrations for activity while denature at low salt. However, since ionic interactions are reduced with the increase in salt concentration of the medium, hydrophobic interactions play major role in stabilizing protein structure at higher NaCl concentrations. Since Pol λ has been found to be involved in repair of UV-B induced photoproducts and high salinity generated double strand breaks in plant genome, we reasoned that the protein structure probably remain somewhat stable under UV-B stress or higher intracellular NaCl level for the damage repair activity. Based on this, we studied the possibility of unfolding and aggregation of Pol λ *in vivo* following exposure to UV-B light and high salt treatment. The *in vivo* aggregation studies have indicated partial unfolding and probable aggregation of Pol λ lacking the BRCT domain (Del 2 fragment) after UV-B exposure of the protoplasts, while high salinity induced unfolding Pol λ, deficient in both the BRCT domain and the Ser-Pro rich region (Del 3 fragment) was also observed as evidenced by the appearance of these proteins in the insoluble fraction.

It is now becoming clear that posttranslational modification is an essential constituent of the DNA repair mechanism. Chaperone mediated folding and proteasome-mediated degradation has been found to regulate DNA repair protein functions [54–55]. Numerous studies have suggested key role of HSP90, the essential eukaryotic molecular chaperone, in DNA damage response signaling pathway via regulation of proper folding and stability of client proteins of the DNA repair machinery [56]. HSP90 has been identified as a crucial regulator of DNA Pol η (a Y family DNA polymerase involved in mutagenic TLS), helping Pol η accumulation at stalled replication forks via regulation of stability of the client protein in UV-irradiated cells [44]. HSP90 facilitates the folding of REV1, another Y family DNA Pol involved in TLS, into a stable and functional form, helping REV1 interaction with monoubiquitinated proliferating cell nuclear antigen (PCNA) for mutagenic translesion DNA synthesis [43]. Based on this information and considering the function of Pol λ in repair of oxidative DNA damage and DSBs in *Arabidopsis* [19–21], we next investigated whether Pol λ interacts with HSP90. Immunoprecipitation studies establish that Pol λ is a client of HSP90 (Fig 8A and 8B). HSP90 interacted directly and specifically with Pol λ *in vivo* and at the endogenous level. GDA treatment was able to reduce Pol λ level considerably in *Arabidopsis* seedlings (Fig 8E). In addition, our preliminary results of artificial mircoRNA (amiRNA) mediated transient knockdown of four cytosolic *Arabidopsis* HSP90 genes (*AtHSP901-4*) in *Arabidopsis* leaf mesophyll cell protoplasts have indicated reduced endogenous Pol λ protein level (unpublished data). Together, these results indicate that reduction of Pol λ protein level in *Arabidopsis* seedlings due to HSP90 depletion by GDA treatment or amiRNA mediated knockdown of HSP90 possibly linked with enhanced proteasomal degradation of Pol λ molecules. This notion was supported by the observation of compromised Pol λ degradation in *Arabidopsis* seedlings in presence of the proteasomal inhibitor MG123 (Fig 8F). In addition, treatment of *Arabidopsis* seedlings with the translational inhibitor cycloheximide in the absence or presence of GDA indicated that GDA treatment reduced half-life of Pol λ in *Arabidopsis* seedlings (Fig 8G). These observations, although indicates the possibility that HSP90 may regulate the stability of Pol λ, but raises the important question as to whether HSP90 regulates folding of Pol λ into a stable form (s) to facilitate its interaction with other partner (s) for recruitment of Pol λ molecule into the site of DNA damage in the nucleus. Therefore, further experimental verifications are required to establish the functional significance of HSP90 interaction with Pol λ to unveil HSP90 and proteasome-mediated posttranslational regulation of Pol λ in plants to understand the structure-function stability of Pol λ under abiotic and genotoxic stresses and shed more light on role of HSP90 in DNA damage response in plants.

## Supporting Information

S1 Fig Schematic representation of domain and sub-domain organization of *Arabidopsis thaliana* DNA Pol. (TIF)

S2 Fig Purification of recombinant Pol λ and Del 1 proteins. (TIF)

S3 Fig Purification of recombinant Del 2 and Del 3 protein fragments. (TIF)

S4 Fig I_337_/ I_350_ plot of UV-B irradiated recombinant Pol λ and its deletion mutants. (TIF)

S5 Fig Tryptophan fluorescence spectra of high salt treated purified recombinant Pol λ and Del 1 proteins. (TIF)

S6 Fig Tryptophan fluorescence spectra of high salt treated purified recombinant Del 2 and Del 3 proteins. (TIF)

S7 Fig Quenching of intrinsic tryptophan fluorescence of recombinant Pol λ. (TIF)

S8 Fig Amide I FT-IR spectra of recombinant full length Pol λ and its N terminus deletion fragments. (TIF)

S9 Fig Changes in the secondary structural elements of Pol λ and its N terminus deletion mutants after UV-B and high salt stress by FT-IR spectroscopy. (TIF)

S10 Fig *In vitro* DNA polymerase activity assay using purified recombinant Pol λ and its N-terminal deletion mutants. (TIF)

S11 Fig Expression constructs used for *Agro*-infiltration of tobacco or transformation of *Arabidopsis*. (TIF)

S12 Fig Ectopic expression of AtHSP90 in tobacco leaves. (TIF)

S1 File Supplementary materials. (DOC)

S1 Table Changes in the secondary structure compositions (in percentage) of full length and the N terminus truncated forms of recombinant purified Pol λ after UV-B and high salt stress. (DOC)
