# Supplementary figures and images for: Understanding the Physical and Molecular Basis of Stability of Arabidopsis DNA Pol λ under UV-B and High NaCl Stress
Source: PLoS One. 2015 Jul 31;10(7):e0133843. doi: 10.1371/journal.pone.0133843 (PMC4521722; doi:10.1371/journal.pone.0133843)

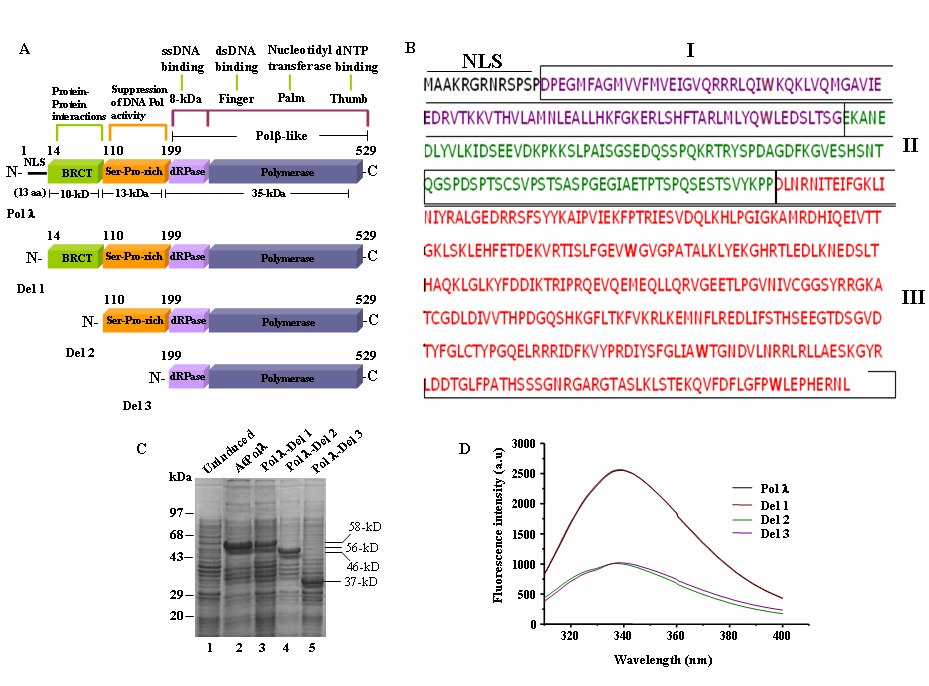

Supplement: S1 Fig — (TIF) [file pone.0133843.s001.tif]

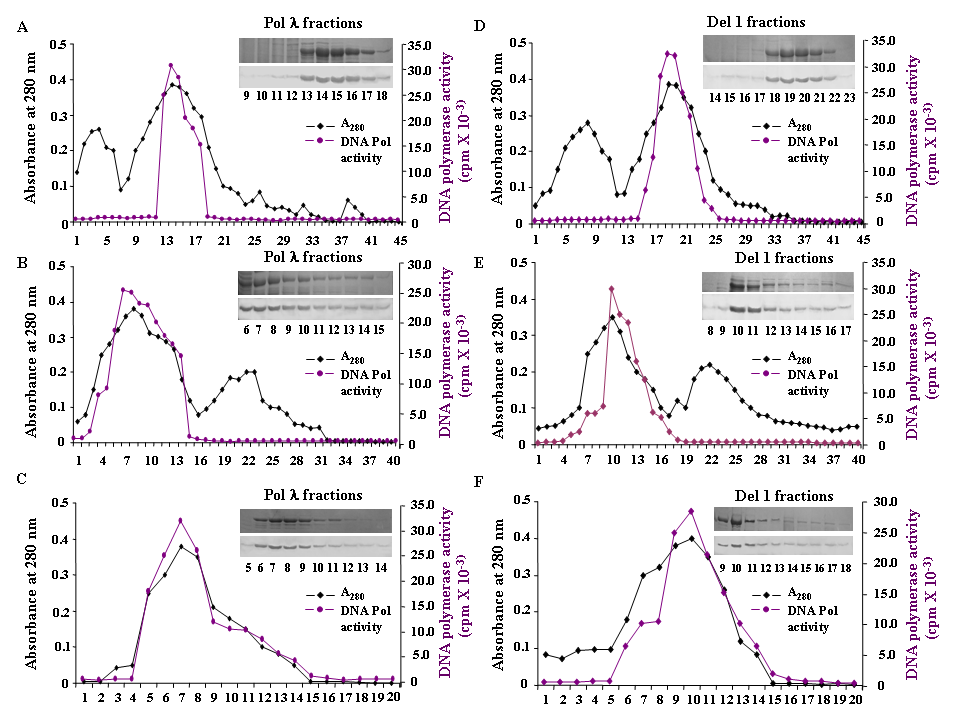

Supplement: S2 Fig — (TIF) [file pone.0133843.s002.tif]

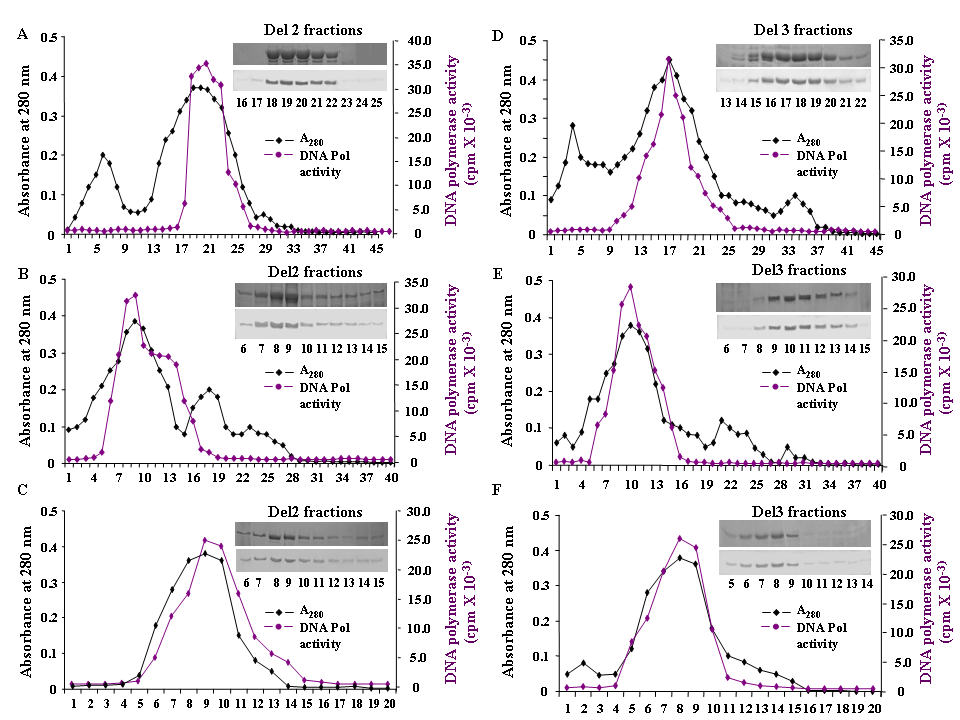

Supplement: S3 Fig — (TIF) [file pone.0133843.s003.tif]

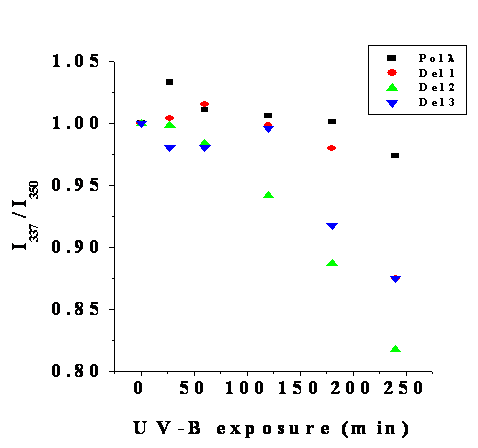

Supplement: S4 Fig — (TIF) [file pone.0133843.s004.tif]

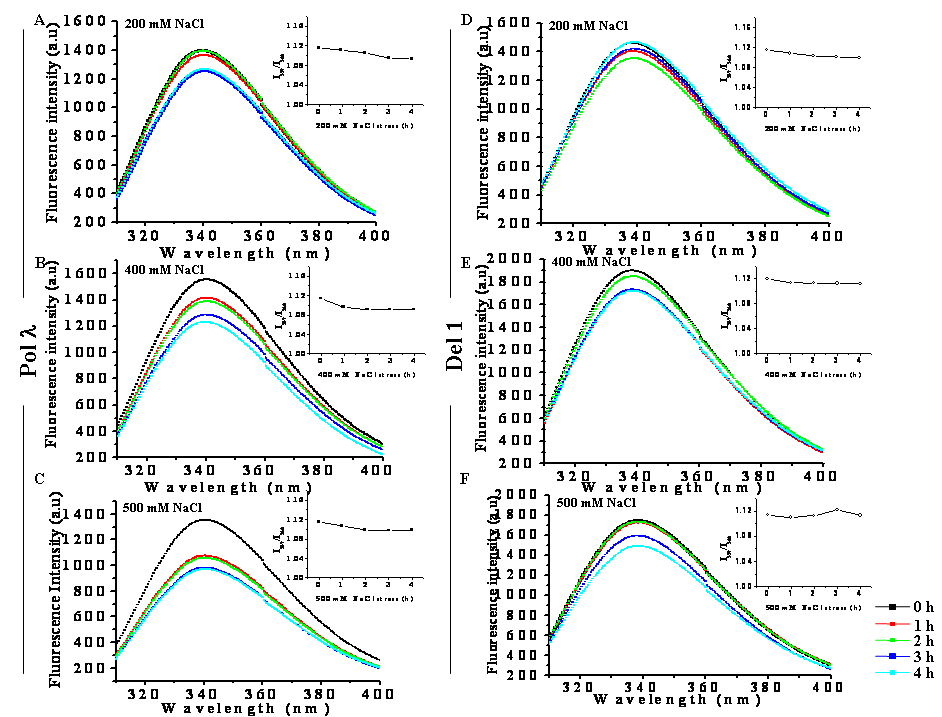

Supplement: S5 Fig — (TIF) [file pone.0133843.s005.tif]

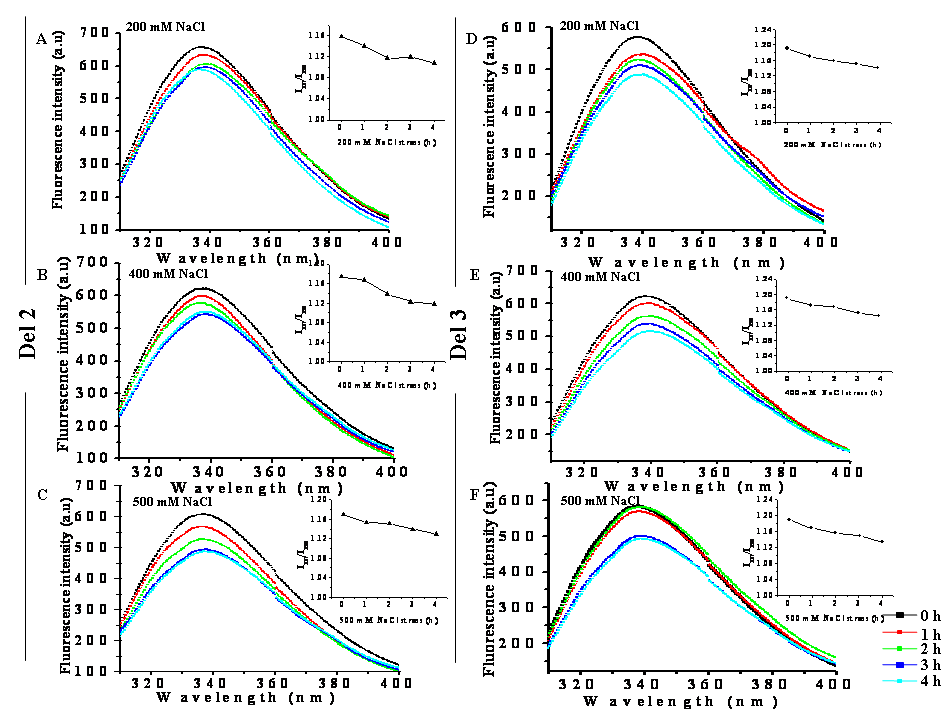

Supplement: S6 Fig — (TIF) [file pone.0133843.s006.tif]

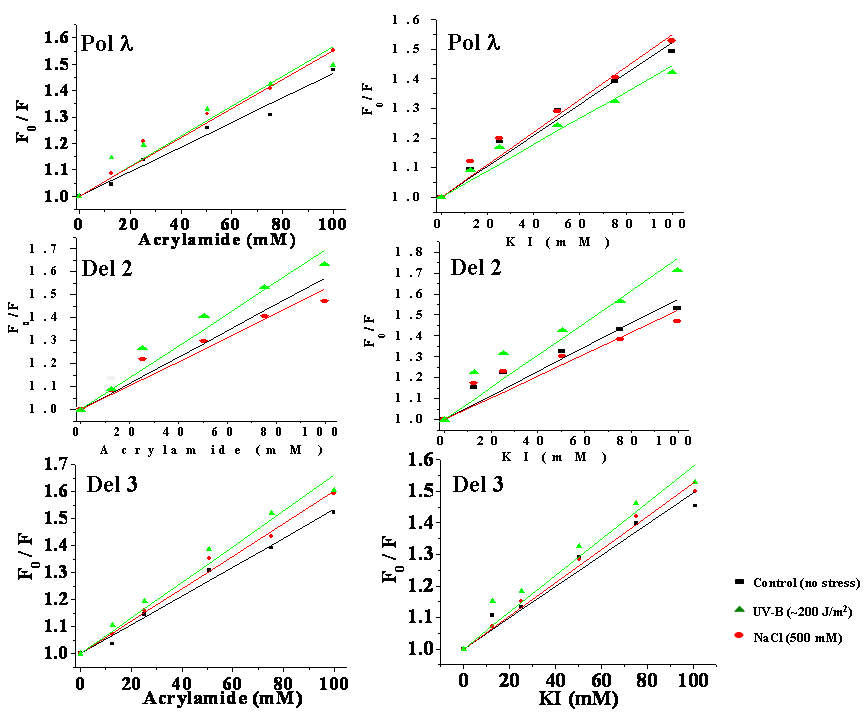

Supplement: S7 Fig — (TIF) [file pone.0133843.s007.tif]

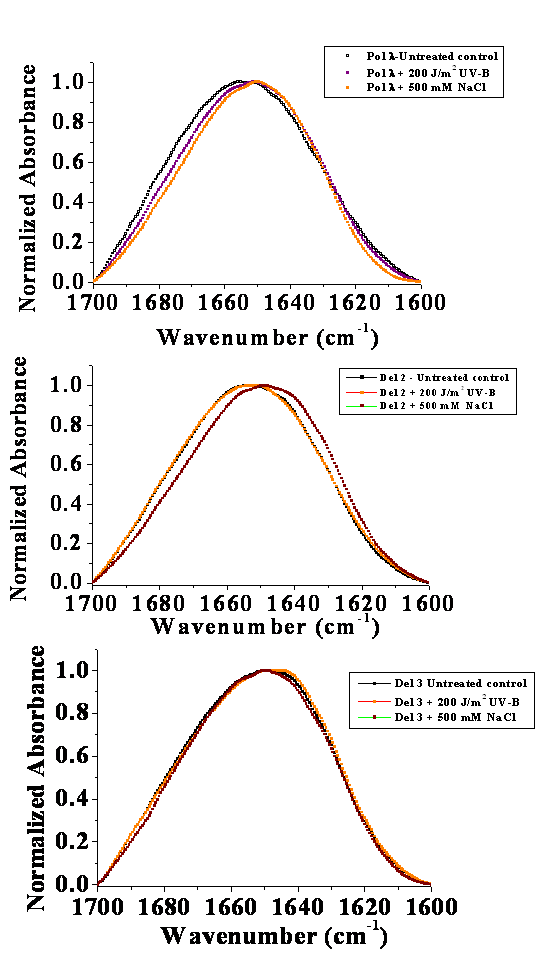

Supplement: S8 Fig — (TIF) [file pone.0133843.s008.tif]

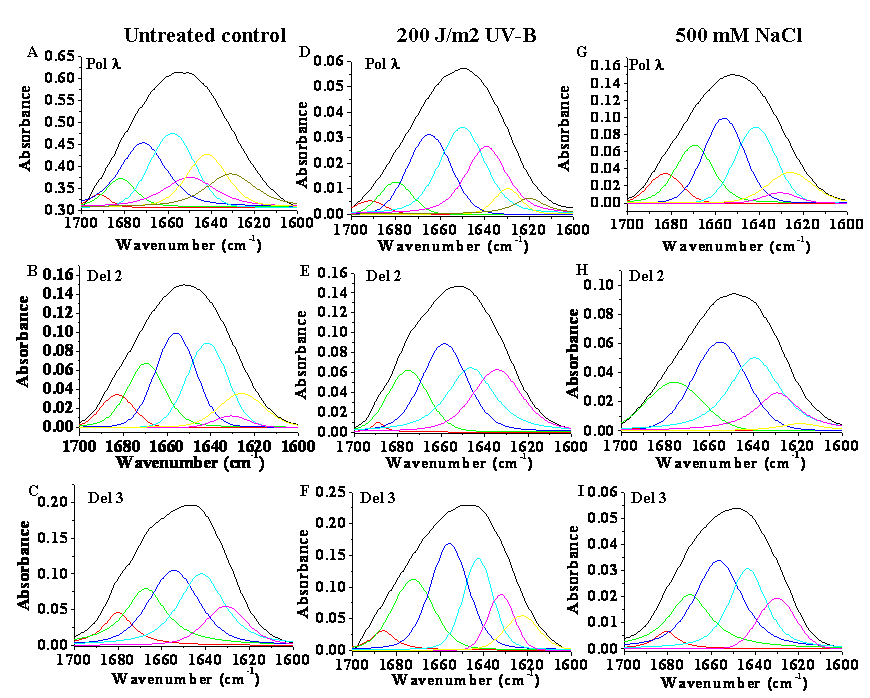

Supplement: S9 Fig — (TIF) [file pone.0133843.s009.tif]

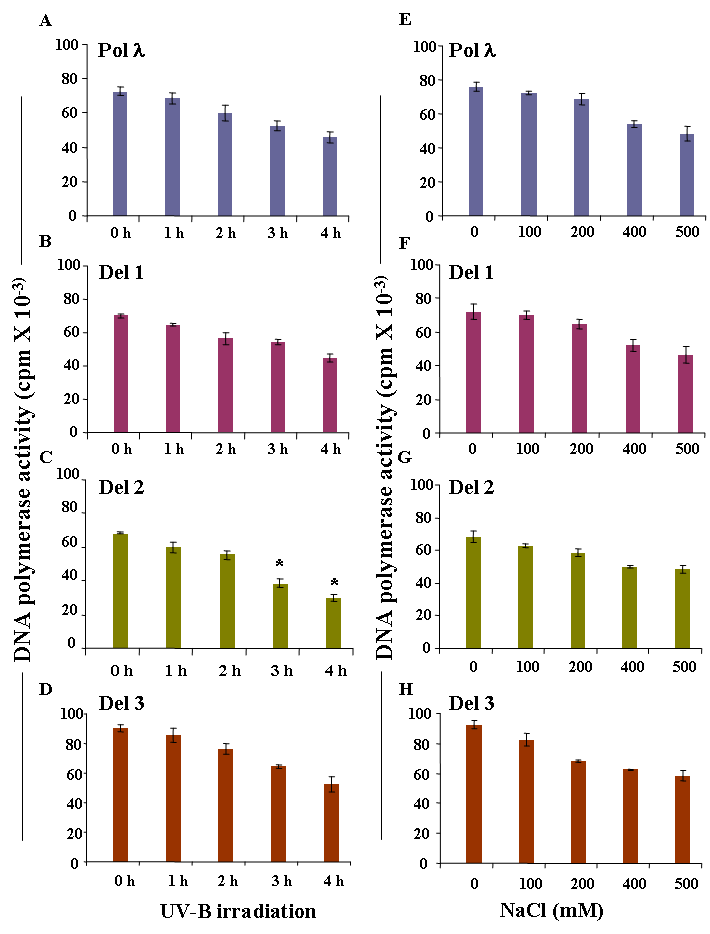

Supplement: S10 Fig — (TIF) [file pone.0133843.s010.tif]

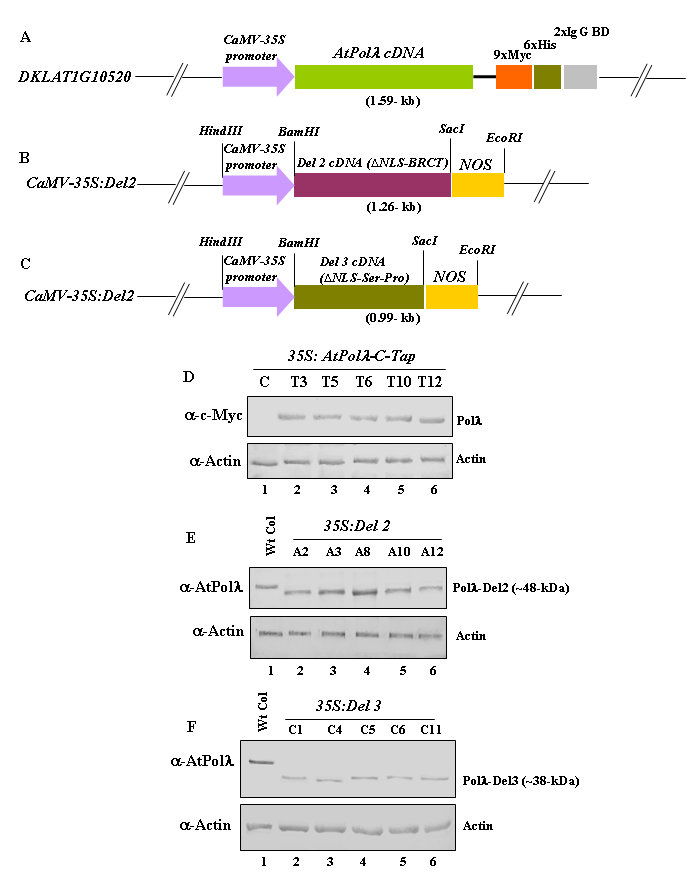

Supplement: S11 Fig — (TIF) [file pone.0133843.s011.tif]

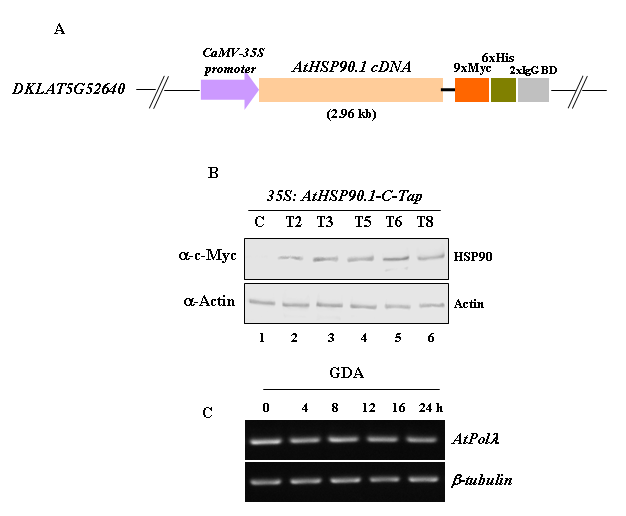

Supplement: S12 Fig — (TIF) [file pone.0133843.s012.tif]
