# Supplementary material for: Understanding the Physical and Molecular Basis of Stability of Arabidopsis DNA Pol λ under UV-B and High NaCl Stress
Source: PLoS One. 2015 Jul 31;10(7):e0133843. doi: 10.1371/journal.pone.0133843 (PMC4521722; doi:10.1371/journal.pone.0133843)
Supplement: S1 File — (DOC) [file pone.0133843.s013.doc]

**Supporting Materials**

**UV-B irradiation of Arabidopsis and tobacco mesophyll cell protoplasts and salinity treatment of Arabidopsis seedlings**

~1 ml of floating protoplast suspension in W5 solution (at the density of ~1.5 X 10^5^/ml) in a 35 mm Petri dish was irradiated with UV-B light (~200 J/m^2^) for various time points. Irradiated protoplast samples were collected after specific time intervals, immediately frozen in liquid nitrogen and stored at -80ºC. UV-B irradiation of seedlings and protoplasts were performed in a growth chamber equipped with a UV-B light source (UV-B bulb, Philips) which radiates at a wavelength of >280 nm with a high peak at 312 nm [1]. UV-B dose was so chosen that it produced physiological effects without drastically damaging or killing the protoplasts. UV-B dose between 150-200 J/m^2^ was found to clearly induce the expression of some well known UV-inducible genes including chalcone synthase (*CHS*), encoding enzyme that catalyzes first committed step in flavonoid biosynthesis and also Polλ, as described in previous studies [1,2] without drastically affecting protoplast growth (data not shown). UV-B dose beyond 300 J/m^2^ significantly reduced the survival of protoplasts.

For analyzing the possibility of high salinity mediated aggregation of Pol λ, 7-days old transgenic *atpolλ-1^Polλ-Del2ox^*, *atpolλ-1^Polλ-Del3ox^* seedlings were grown on MS-agar medium or leaves from four-week old tobacco plants (transiently expressing full length C terminal Tap tagged *AtPolλ* under the constitutive 35S CaMV promoter) were transferred to one-half-strength liquid MS medium containing different concentrations of NaCl. Seedlings or leaves were incubated for 8 h at room temperature and then immediately frozen in liquid nitrogen and stored at -80ºC for protein isolation and immunoblotting experiments.

**Protoplast Preparation from Arabidopsis and tobacco leaf mesophyll cells**

Protoplast isolation from *Arabidopsis* and tobacco (*Nicotiana benthamiana*) leaves were carried out following Salvucci and Anderson (1987) and Kim and Somers (2010) with minor modifications [3,4] . All procedures were carried out in a clean hood. Protoplasts were isolated from 6 to 8 leaves of 25 to 28-days old *Arabidopsis* and tobacco plants maintained under long day conditions (16 h L/8 h dark) at 23ºC and 28ºC respectively by enzymatic digestion. The leaves were briefly sterilized with 70% ethanol for 30 s and then rinsed in sterile water for two-three times. The adaxial leaf surface was briefly abraded with a sandpaper and then soaked in ~7 mL of an enzyme solution (400 mM mannitol, 20 mM KCl, 20 mM MES-KOH [pH 5.7], 10 mM CaCl_2_, 1% Cellulase R10, 0.5% Macerozyme R10, and 0.1% bovine serum albumin) in a 60-mm-diameter Petri dish. The enzymatic digestion of leaf sample was carried out in the dark, with gentle shaking on rocker (35 rpm) for about two and a half hours at room temperature (25ºC). The released protoplasts were filtered through a sterile 100 mm nylon mesh and then transferred into round bottom 15 mL culture tubes containing 5 mL of W5 solution (154 mM NaCl, 125 mM CaCl_2_, 5 mM KCl, 1.5 mM MES-KOH [pH 5.7], and 5 mM glucose). Protoplasts were harvested by centrifugation at 100 g for 5 min at room temperature (25ºC). After carefully removing the supernatant, the protoplast pellet was resuspended in 2 mL of ice-cold W5 solution and kept on ice for 30 min. Protoplasts were centrifuged and resuspended in 400 mM mannitol, 15 mM MgCl2, and 4 mM MES-KOH (pH 5.7), at a concentration of 1 to 1.5 X 10^5^ mL^-1^.

**Infiltration of tobacco leaves with *Agrobacteria***

A C-terminal TAP-tagged full length *AtPolλ* cDNA clone in a T-DNA vector was transformed into *Agrobacterium tumefaciens* strain GV3101, resuspended in *Agro*-infiltration medium (10 mM MgCl_2_, 150 μM acetosyringone and 10 mM MES pH 5.7) to A_600_ = 0.8, and co-infiltrated with 35S:p19 construct in the abaxial side of leaves of 25-28-days old tobacco (*Nicotiana* *benthamiana*) plants. About 36 h post infiltration, the leaves were subjected to different concentrations of NaCl for 8 h. The untreated control and salt treated leaves were ground to fine powder in liquid nitrogen, protein extracted with ice-cold total protein extraction buffer (50 mM Tris–HCl pH 7.5, 100 mM NaCl, 2.0 mM EDTA, 5 mM MgCl2, 400 mM sucrose, 5% glycerol, 1 mM PMSF, 5 μg/ml Leupeptin, 1 μg/ml Aprotinin, 1 μg/ml Pepstatin, 5 μg/ml Antipain, 5 μg/ml Chymostatin, 2mM NaVO3, 2mM NaF and 50 μM MG132). After centrifugation, both supernatant and pellet fractions were subjected to immunoblotting for detection of the presence of AtPolλ.

**Protein extraction and immunoblotting**

Total protein extracts from *Arabidopsis* seedlings or control and *Agro*-infiltrated tobacco leaves were carried out from approximately 100 mg of plant tissues by following the method described previously [1]. Protein concentrations in the supernatant (soluble) and pellet (insoluble) fractions were determined by Bradford assay (Bradford, 1976) using bovine serum albumin (Fraction V, Sigma) as a standard [5]. Protein extraction from control or transfected protoplasts was carried out by following Yoo et al., 2007 [6]. The protoplast suspension in W5 solution was first centrifuge at 1200 rpm for 5 min in a swing-out rotor at 4ºC and the supernatant was carefully removed. The protoplasts were then resuspended in 25 μl of freshly prepared protoplast lysis buffer (25 mM Tris–phosphate pH 7.8, 1 mM DTT, 10% [vol/vol] glycerol, 1% [vol/vol], Triton X-100, 1 mM PMSF, 5 μg/ml Leupeptin, 1 μg/ml Aprotinin, 1 μg/ml Pepstatin, 5 μg/ml Antipain, 5 μg/ml Chymostatin, 2mM NaVO3, 2mM NaF and 50 μM MG132) and frozen in liquid nitrogen. After thawing on ice, the samples were centrifuged at 12,000 rpm for 10 min at 4°C. Both the clear supernatant and pellet fractions were used for Western blotting experiments for detection of AtPolλ in soluble and insoluble forms. The supernatant and the pellet fractions were mixed with 4X loading buffer (DTT), incubated at 95°C for 2 min and then protein samples were resolved on a 10% SDS-PAGE for immunoblotting as described previously [1].

**Immunoprecipitation**

For immunoprecipitation of DNA Pol λ and co-immunoprecipitation of HSP90, tissues were ground and resuspended in IP buffer (50 mM Tris·Cl, pH 7.5/150 mM NaCl/0.5% NP-40/1 mM PMSF/1 µg/ml leupeptin/1 µg/ml aprotinin/1 µg/ml pepstatin), clarified by spinning at 16,000 rpm (15 min at 4ºC). Protein extracts (1 mg) were then incubated with affinity purified anti-AtPolλ polyclonal antibody (1:250 dilution) or pre-immune serum (1:1000 dilution) overnight at 4ºC with gentle agitation. Immune complexes were collected by incubation with 50 µl of protein A-agarose (Sigma) at 4ºC for 4 h, followed by brief centrifugation. Immune complexes were washed three times for 5 min in 1 ml of IP buffer, resuspended in 50 µl of 2× SDS/PAGE sample buffer, briefly heated, and subjected to SDS/PAGE and immunoblotting. Immunoblot detection using affinity purified anti-AtPolλ polyclonal antibody was described previously [1]. For HSP90 immunoblotting, protein samples were transferred to PVDF membrane (Bio-Rad) and incubated with affinity purified anti-HSP90 antibody (Agrisera, 1:1000 dilution) for overnight at 4ºC. The membranes were developed using peroxidase-conjugated secondary antibody (1:3,000–5,000) (GE Healthcare) by enhanced chemiluminescence (SuperSignal West Pico Chemiluminescent substrate; Pierce Biotechnology). Ten-day-old *Arabidopsis* whole seedlings grown under 16 h light/8 h dark condition on MS plates or *Nicotiana benthamiana* leaves 3 d after *Agro*-infiltration were subjected to *in vivo* protein cross-linking as previously described [7].

**Expression, and purification of recombinant full length and N terminus deletion fragments of DNA Pol λ**

The full length 1.59 kb *AtPolλ* cDNA was amplified by PCR and cloned in frame at the *BamHI-SacI* sites of pQE30 expression vector (Qiagen), while the N terminus truncated forms of *Pol λ* cDNA were amplified by PCR using pQE30:*AtPolλ* as a template. The N-terminus deletion mutants of AtPolλ including Del 1 (lacks the N-terminus nuclear localization sequence - NLS, 1-13 aa residues), Del 2 (lacks the N-terminus NLS and BRCT domains, 1-109 aa residues) and Del 3 (lacking the N-terminus NLS, BRCT and Ser-Pro rich SDPA domains, 1-198 aa residues) were described previously [8]**.** The pQ30:*AtPolλ* recombinant plasmids encoding either the full-length ~60-kD protein or the N terminus deletion fragments, Del 1 (~56-kD), Del 2 (~48-kD) and Del 3 (~40-kD) of Pol λ were expressed in *E. coli* cells (M15, pREP 4) (Qiagen) as N-terminus 6X-His tagged fusion proteins. The recombinant protein expression was induced was induced with 1 mM isopropylthio-β-galactoside (IPTG) for 18 h at 37ºC and isolated from 2 L of bacterial culture containing 100 mg/mL ampicillin and 25 mg/mL kanamycin respectively. Cells were harvested by centrifugation at 5000 rpm at 4ºC for 30 min and bacterial pellets were resuspended in a 30 mL lysis buffer containing 50 mM Tris-HCl, pH 7.5, 300 mM NaCl, 5 mM MgCl_2_, 10 mM β-mercaptoethanol and 1 mM PMSF. Lysozyme was added to a final concentration of 1mg/ml and cell lysate was then incubated on ice for 1 h. The lysates were then briefly sonicated (6 cycles of 1 min each) to destroy cell membranes and reduce viscosity, followed by centrifugation at 15000 rpm at 4ºC for 10 min. After centrifugation solubility of his-tagged recombinant proteins were checked in 12% SDS- PAGE. The pellet fractions containing the major part of the expressed proteins were solubilized in a buffer containing urea (8 M urea, 50 mM Tris-HCl, pH 7.5, 0.5 M NaCl, 10 mM β-ME, 1 mM PMSF), following by gentle swirling of the sample on a rotary shaker for 45 min at room temperature (25ºC). Solubilized samples were centrifuged at 15,000 rpm for 15 min. The supernatant fraction containing the recombinant protein was then renatured by removing urea following ‘step-dialysis’ procedure in the similar buffer containing 7, 6, 5, 4, 3, and 2 M urea for 2 h each at room temperature and finally in urea-free protein refolding buffer (50 mM Tric-HCl, pH 7.5, 50 mM NaCl, 5% glycerol, 1 mM β-ME, 1 mM PMSF) for 12 h at 4ºC with three changes. Dialyzed expressed protein sample was concentrated by lyophilization and ~1 mL of protein sample was then applied to Sephacryl S300-HR (GE Healthcare) size exclusion column (1.0 x 50 cm, BioRad)**,** followed by elution in buffer containing 10 mM Tric-HCl, pH 7.5, 100 mM NaCl, 1 mM EDTA, 1 mM β-ME and 1 mM PMSF. Main peak fractions with DNA polymerase activity were analysed resolving in 10% SDS-PAGE and Western blotting using anti-AtPolλ antibody (1:500 k dilution) described previously [1], pooled, concentrated and then loaded on to Sephacryl S200-HR (GE Healthcare) size exclusion column (1.0 x 50 cm, BioRad)**.** The active peak fractions were then pooled and dialysed in a buffer containing 10 mM Tric-HCl, pH 7.5, 1 mM β-ME and 1 mM PMSF to reduce salt to below 50 mM. The dialyzed sample (~10 mL) was loaded on to a HiTrap Capto Q column (1 mL, GE Healthcare) and directly eluted in presence of 100 mM NaCl. The major active peak fractions were dialyzed against protein refolding buffer and stored in aliquots at -70 °C (S2 Fig. and S3 Fig).

**Measurement of DNA polymerase activity**

DNA polymerase activity assay was carried out using purified full length and N terminus deletion versions of AtPolλ by following the method described previously [9]**.** Briefly, *in vitro* DNA polymerase activity assay was preformed by measuring the incorporation of [3H]dTMP in 10% trichloroacetic acid insoluble fractions by following the protocol as described previously [9]. Reactions were performed in a final volume of 50 ll containing 50 mM Tris–Cl, pH 7.5, 5 mM MgCl2, 10 mM DTT, 0.05 mM of each of dATP, dCTP, dGTP and 1 lCi of [3H]dTTP (Sp. Ac. 89 Ci/mmol) and 20 lg/ml of activated calf thymus DNA or poly(dA)-oligo(dT) as template–primer. Reaction mixtures were incubated at 37ºC for 30 min and then terminated by the addition of 5 ll of carrier DNA (sheared salmon sperm DNA, 10 mg/ml) and 2 ml of chilled 10% TCA containing 100 mM sodium pyrophosphate. After proper mixing reactions were kept on ice for 30 min. The precipitates were collected on prewashed (with sterile H_2_O) GF/C filters (Whatman) in a vacuum filtration device (Millipore). Filters were then washed with ice-cold 2% TCA twice and once with 90% ethanol. Filters were dried under heat lamp and radioactivity of each filter was measured in a Liquid Scintillation Counter (Beckman L5) using scintillation fluid.

***In vivo* aggregation study**

The possibility of *in vivo* aggregation of Pol λ was studied in the absence or presence of UV-B or high salt stress, based on the idea that unfolded or aggregated protein will become insoluble and exist in the pellet fraction of the total soluble protein extracts. Intact protoplasts from wild-type *Arabidopsis* (expressing the endogenous *Pol λ*) and tobacco (*Nicotiana benthamiana*) leaf mesophyll cell protoplasts (transiently expressing C-terminal TAP-tagged *AtPolλ* under CaMV-35S promoter, S10A Fig.) were exposed to UV-B light (~200 J/m^2^) in the dark for various time points and protein extracts were prepared. For high salt stress, 7-days-old wild-type *Arabidopsis* seedlings or *Agro*-infiltrated tobacco leaves (transiently expressing AtPolλ) were subjected to increasing concentrations of NaCl (0-300 mM) treatment for 8 h at room temperature followed by protein extraction. Protein samples from the soluble (supernatant) and insoluble (pellet) fractions of protoplasts or seedling tissues were analysed by SDS-PAGE and immunoblotting using anti-AtPolλ (for *Arabidopsis*) or anti-Myc (for tobacco) antibodies, respectively to determine whether Pol λ could be effectively found in an aggregated state *in vivo* (Fig. 7G-J). The extent of aggregation that occurred for specific proteins was assumed by the relative amount of target protein recovered in the soluble and insoluble fractions as measured by immunoblot analysis. In case of protoplast treatment, to minimize protein aggregation after lysis, protoplasts were lysed in pre-chilled tubes and the tubes placed at a constant temperature of 23°C. This procedure cooled the solutions during lysis. In addition, the use of the detergent, Triton X-100, caused rapid lysis of the chloroplast and protoplast and eliminated the possible interactions between soluble proteins and membranes. Control experiments showed that ~92-95% of the protoplasts remained intact prior to lysis based on the amount of soluble protein present in the supernatant under control or after stress treatment.

To analyse the effect of deletion of N-terminal domains on the aggregation of DNA Pol λ in presence of UV-B or high salt *in vivo*, immunoblotting experiments were carried out using soluble and insoluble protein extracts from control and UV-B irradiated leaf mesophyll cell protoplasts from stable transgenic *atpolλ-1* mutant plants (devoid of *AtPolλ* expression) expressing *Pol λ-Del2* and *Pol λ-Del3* cDNA fragments driven by the constitutive cauliflower mosaic virus (CaMV) 35S promoter (S10B-F Fig.). For salt stress, 7-days-old transgenic *atpolλ-1^Pol λ-Del2^* and *atpolλ-1^Pol λ-Del3^* seedlings were exposed to increasing concentration of NaCl at room temperature and then protein extracts were prepared. Immunoblotting was carried out using the soluble and insoluble protein fractions using anti-AtPolλ antibody (Fig. 7K-N, upper and lower panels).

**RNA isolation and transcript profile analysis by Semi quantitative RT-PCR**

Total RNA was isolated from ~100 mg of seedling tissues using RNeasy plant minikit (Qiagen) following manufacturer’s instruction. RNA samples were treated with DNase I (RNase free, Roche) according to manufacturer’s protocol to remove genomic DNA contaminations. First strand cDNA was synthesized from 1 μg of total RNA sample using AMV first strand cDNA synthesis kit (Roche) following manufacturer’s instructions. Transcript profile was analysed by semi quantitative RT-PCR following the protocol described previously [8].

**Supplementary Literature Cited**

1. Roy S, Choudhury SR, Singh SK, Das KP (2011) AtPolλ, A Homolog of Mammalian DNA Polymerase λ in Arabidopsis thaliana, is Involved in the Repair of UV-B Induced DNA Damage Through the Dark Repair Pathway. Plant Cell Physiol 52: 448-467.

2. Amoroso A, Concia L, Maggio C, Raynaud C, Bergounioux C, et al. (2011) Oxidative DNA Damage Bypass in Arabidopsis thaliana Requires DNA Polymerase λ and Proliferating Cell Nuclear Antigen 2. The Plant Cell 23: 806-822.

3. Salvucci ME, Anderson JC (1987) Factors Affecting the Activation State and the Level of Total Activity of Ribulose Bisphosphate Carboxylase in Tobacco Protoplasts. Plant Physiol 85: 66-71.

4. Kim J, Somers DE (2010) Rapid Assessment of Gene Function in the Circadian Clock Using Artificial MicroRNA in Arabidopsis Mesophyll Protoplasts. Plant Physiol 154: 611-621.

5. Bradford MM (1976) A rapid and sensitive method for the quantitation of microgram quantities of protein utilizing the principle of protein-dye binding. Analytical Biochemistry 72: 248-254.

6. Yoo S-D, Cho Y-H, Sheen J (2007) Arabidopsis mesophyll protoplasts: a versatile cell system for transient gene expression analysis. Nat Protocols 2: 1565-1572.

7. Rohila JS, Chen M, Cerny R, Fromm ME (2004) Improved tandem affinity purification tag and methods for isolation of protein heterocomplexes from plants. Plant J 38: 172-181.

8. Roy S, Choudhury SR, Sengupta DN, Das KP (2013) Involvement of AtPolλ in the Repair of High Salt- and DNA Cross-Linking Agent-Induced Double Strand Breaks in Arabidopsis. Plant Physiol 162: 1195-1210.

9. Roy S, Roy Choudhury S, Mukherjee SK, Sengupta DN (2007) Tobacco proliferating cell nuclear antigen binds directly and stimulates both activity and processivity of ddNTP-sensitive mungbean DNA polymerase. Archives of Biochemistry and Biophysics 468: 22-31.

**Supplementary Figure Legends**

**S1 Fig.** Schematic representation of domain and sub-domain organization of *Arabidopsis thaliana* DNA Pol λ (AtPolλ, GenBank accession: ADM33939) and its N terminus deletion fragments. (A) Linear diagram indicating the different domains which constitute the full-length Pol λ protein: BRCT domain, Ser-Pro rich region, 8 kDa domain and the polymerase catalytic core domain, comprising of Fingers, Palm, and Thumb sub-domains, with the functions of different regions. The amino acid numbers for the full length and N terminus deletion fragments of AtPolλ are indicated. NLS indicates nuclear localization signal. (B) Deduced amino acid sequence of AtPolλ. The important domains are shown within the boxes: box I represents BRCT domain, box II – Ser-Pro rich region and box III – the C terminus conserved catalytic core polymerase domain respectively. (C) Coomassie Blue staining of 10% SDS-Polyacrylamide gel showing separation of control non-induced (lane 1) and IPTG-induced (lanes 2-5) extracts of *E. coli* M15 (pREP4) cells expressing the full length AtPolλ or the N terminus deletion fragments as 6X-His tagged proteins. ~25 μg protein extracts were loaded in each lane. The electrophoretic migration of the protein bands are indicated on the right side of the gel while positions of molecular weight markers have been shown on the left. (D) Tryptophan fluorescence spectra of purified recombinant full length and N terminus deletion fragments of AtPolλ, indicating no significant peak shift in the spectra and thus folding property of AtPolλ after deletion of N terminus regions. Reduction in fluorescence intensity of Del 2 and Del 3 proteins were due to deletion of tryptophan residues at the N terminus region of AtPolλ.

**S2 Fig.** Purification of recombinant Pol λ and Del 1 proteins. (A-C) Protein elution and DNA polymerase activity profiles of recombinant full length Pol λ from Sephacryl S-300 column (A), Sephacryl S-200 column (B) and HiTrap Capto Q column (C), respectively. The embedded gel images in (A-C) correspond to Coomassie blue stained 10% SDS-PAGE of the indicated column fractions (upper panel) and protein gel blot analysis of the corresponding fractions with anti-AtPolλ antibody (1:500 dilution) (lower panel). Fraction numbers are indicated at the bottom of each lane. (D-F) Protein elution and DNA polymerase activity profiles of recombinant Del 1 fragment from Sephacryl S-300 column (D), Sephacryl S-200 column (E) and HiTrap Capto Q column (F). The embedded gel images in (D-F) correspond to Coomassie blue stained 10% SDS-PAGE of the indicated column fractions (upper panel) and protein gel blot analysis of the corresponding fractions with anti-AtPolλ antibody (1:500 dilution) (lower panel). Fraction numbers are indicated at the bottom of each lane.

**S3 Fig.** Purification of recombinant Del 2 and Del 3 protein fragments. (A-C) Protein elution and DNA polymerase activity profiles of recombinant Del 2 fragment from Sephacryl S-300 column (A), Sephacryl S-200 column (B) and HiTrap Capto Q column (C). The embedded gel images in Figures A-C correspond to Coomassie blue stained 10% SDS-PAGE of the indicated column fractions (upper panel) and protein gel blot analysis of the corresponding fractions with anti-AtPolλ antibody (1:500 dilution) (lower panel). Fraction numbers are indicated at the bottom of each lane. (D-F) Protein elution and DNA polymerase activity profiles of recombinant Del 3 fragment from Sephacryl S-300 column (D), Sephacryl S-200 column (E) and HiTrap Capto Q column (F). The embedded gel images in Figures (D-F) correspond to Coomassie blue stained 10% SDS-PAGE of the indicated column fractions (upper panel) and protein gel blot analysis of the corresponding fractions with anti-AtPolλ antibody (1:500 dilution) (lower panel). Fraction numbers are indicated at the bottom of each lane.

**S4 Fig.** I_337_/ I_350_ plot with the time of UV-B exposure to monitor the UV-B induced conformational change of purified recombinant Pol λ and its N-terminal deletion fragments.

**S5 Fig.** Tryptophan fluorescence spectra of high salt treated purified recombinant Pol λ and Del 1 protein. (A-C) 0.05 mg/mL of purified full length recombinant Pol λ or (D-F) Del1 protein in a final volume of 600 μL of 50 mM Tric-HCl buffer, pH 7.5 (containing 1 mM β-ME and 1 mM PMSF) were subjected to 200, 400 and 500 mM NaCl treatment for the indicated time points at 25ºC. Tryptophan fluorescence spectra of protein samples were measured using excitation wavelength of 295 nm. The emission wavelengths were set in the range between 300 to 400 nm with the emission scan speed of 240 nm/min. Inset images indicate ratio of fluorescence intensity at 337 nm to the same at 350 nm plotted as function of NaCl concentration.

**S6 Fig.** Tryptophan fluorescence spectra of high salt treated purified recombinant Del 2 and Del 3 protein fragments. (A-C) 0.05 mg/mL of purified Del 2 or Del 3 protein (D-F) in a final volume of 600 μL of 50 mM Tric-HCl buffer, pH 7.5 (containing 1 mM β-ME and 1 mM PMSF) were subjected to 200, 400 and 500 mM NaCl treatment for the indicated time points at 25ºC. Tryptophan fluorescence spectra of protein samples were measured using excitation wavelength of 295 nm. The emission wavelengths were set in the range between 300 to 400 nm with the emission scan speed of 240 nm/min. Inset images indicate ratio of fluorescence intensity at 337 nm to the same at 350 nm plotted as function of NaCl concentration.

**S7 Fig.** Quenching of intrinsic tryptophan fluorescence of recombinant Pol λ by acrylamide and iodide. Stern-Volmer plot for the quenching of tryptophan fluorescence of untreated control, UV-B irradiated (~200 J/m^2^ UV-B for 4 h at 25ºC) and high salt (500 mM NaCl treatment for 2 h at 25ºC) treated recombinant full length Pol λ, Del 2 and Del 3 proteins in presence of (A-C) acrylamide and (D-F) iodide respectively. 0.05 mg/ml of each of the protein sample in 50 mM Tric-HCl buffer, pH 7.5, containing 1 mM β-ME and 1 mM PMSF was excited at 295 nm with the emission wavelengths of 300-400 nm. Tryptophan fluorescence spectra were measured after every addition of aliquots of acrylamide or potassium iodide respectively.

**S8 Fig.** Amide I FT-IR spectra of recombinant full length Pol λ and its N terminus deletion fragments after UV-B or high salt treatment by FT-IR spectroscopy. (A) Purified recombinant Pol λ, (B) Del 2 and (C) Del 3 protein samples were subjected to UV-B irradiation with a dose of ~200 J/m^2^ for 4 h at 25ºC or incubated in presence of 500 mM NaCl for 2 h at 25ºC. The untreated control, UV-B irradiated or salt treated protein samples were concentrated using microcon filter concentrator device equipped with a 3-kDa cut-off membrane (Millipore) to the final concentration of ~10 mg/ml in 50 mM Tris-HCl buffer, pH 7.5, exchanged with D_2_O for 3-4 times and ~20 μl D_2_O exchanged protein sample was used for FT-IR analysis as described under ‘Materials and Methods’.

**S9 Fig.** Monitoring of changes in the secondary structure of full length and the N terminus truncated forms of recombinant purified Pol λ and its deletion mutants after UV-B and high salt stress by FT-IR spectroscopy. (A-C) Secondary structure compositions of untreated control full length Pol λ (A), Del 2 (B) and Del 3 proteins (C), respectively. (D-F) Secondary structure compositions of UV-B irradiated full length Pol λ (D), Del 2 (E) and Del 3 proteins (F). (G-I) Secondary structure compositions of high salt treated full length Pol λ (G), Del 2 (H) and Del 3 proteins (I). Purified protein samples were subjected to UV-B irradiation with a dose of ~200 J/m^2^ for 4 h at 25ºC or incubated in presence of 500 mM NaCl for 2 h at 25ºC. The untreated control, UV-B irradiated or salt treated protein samples were concentrated using microcon filter concentrator device equipped with a 3-kDa cut-off membrane (Millipore) to the final concentration of ~10 mg/ml in 50 mM Tris-HCl buffer, pH 7.5, exchanged with D_2_O for 3-4 times and ~20 μl of D_2_O exchanged protein samples were used for FT-IR analysis. Curve fitting of the original amide I band was done to compute the percentage of secondary structural elements of the protein. Small curves inside the main curve (black) are generated through multi curve fitting program using Thermo GRAMS AI software.

**S10 Fig.** *In vitro* DNA polymerase activity assay using purified recombinant Pol λ and its N-terminal deletion mutants. (A-D) *In vitro* DNA polymerase activity was carried out using purified recombinant full length Pol λ, Del 1, Del 2 and Del 3 proteins, respectively following exposure to UV-B light for various time points. (E-H) DNA polymerase activity of protein samples after pre-incubation in presence of different concentrations of NaCl for 2 h at room temperature (25°C). The error bars indicate mean value from three independent observations.

**S11 Fig.** Expression constructs used for *Agro*-infiltration of tobacco or transformation of *Arabidopsis*. (A) Schematic representation of *pLIC6-CaMV-35S-AtPolλ-C-Tap* expression construct (DKLAT1G10520.1) used for transient expression of *AtPolλ* cDNA in tobacco (*Nicotiana benthamiana*) leaves by *Agro*-infiltration. (B) and (C) The *pCAMBIA-CaMV-*35S:*AtPolλ-Del2* and *pCAMBIA-CaMV-35S:AtPolλ-Del3* constructs used for expression of Pol λ-Del 2 (deficient in NLS and BRCT domain) and Pol λ-Del 3 (devoid of NLS, BRCT and Ser-Pro domains) proteins in *atpolλ-1* null mutant background (devoid of *AtPolλ* expression) by stable transformation. (D) Analysis of expression of Pol λ protein by immunoblotting using ~40 μg of total protein extracts from tobacco leaves ectopically expressing AtPolλ using anti-c-Myc monoclonal antibody (1:500 dilution) (Santa Cruz Biotechnology). Lane 1 shows non-transformed control while lanes 2-6 represent protein samples isolated from leaves of different tobacco plants used for *Argo*-infiltration (plant numbers are indicated within parentheses). The level of actin protein in similar protein extracts was detected using anti-actin (Actin) monoclonal antibody (Sigma) (lower panel). (E) and (F) Immunoblot analyses of total protein extracts from 7-days-old non-transformed wild-type (lane 1) or transgenic *atpolλ-1* mutant line expressing Pol λ-Del 2 (E) and Pol λ-Del 2 proteins (F) (lanes 2-6). 40 μg of total protein extract was loaded in each lane. Affinity purified anti-AtPolλ polyclonal antibody (1:250 dilution) was used to detect Pol λ protein. The level of actin protein in similar protein extracts was detected using anti-actin (Actin) monoclonal antibody (Sigma) and is shown as a loading control (lower panel). Line numbers are indicated within parentheses. Representative gel images from at least three independent trials are shown.

**S12 Fig.** Ectopic expression of AtHSP90 in tobacco leaves. (A) Schematic representation of *pLIC6-CaMV-35S-AtHSP90.1-C-Tap* expression construct (DKLAT5G52640) used for transient expression of AtHSP90.1 in tobacco (*Nicotiana benthamiana*) leaves by *Agro*-infiltration. (B) Analysis of expression of AtHSP90 by immunoblotting using ~40 μg of total protein extracts from tobacco leaves ectopically expressing AtPolλ using anti-c-Myc monoclonal antibody (1:500 dilution) (Santa Cruz Biotechnology). Lane 1 shows non-transformed control while lanes 2-6 represent protein samples isolated from leaves of different tobacco plants used for *Argo*-infiltration (plant numbers are indicated within parentheses). The level of actin protein in similar protein extracts was detected using anti-actin (Actin) monoclonal antibody (Sigma) (lower panel). (C) Effect of geldanamycin treatment on the expression of Pol λ transcript in *Arabidopsis*. 7-days-old wild-type *Arabidopsis* seedlings were treated with 5 µM geldanamycin for the indicated time points. The endogenous mRNA levels of *AtPolλ* were analysed by semi quantitative RT-PCR (upper panel). *β-tubulin* mRNA levels were measured as an internal control (lower panel).
