# Supplementary material for: Understanding the Physical and Molecular Basis of Stability of Arabidopsis DNA Pol λ under UV-B and High NaCl Stress
Source: PLoS One. 2015 Jul 31;10(7):e0133843. doi: 10.1371/journal.pone.0133843 (PMC4521722; doi:10.1371/journal.pone.0133843)
Supplement: S1 Table — (DOC) [file pone.0133843.s014.doc]

**S1 Table:** Changes in the secondary structure compositions (in percentage) of full length and the N terminus truncated forms of recombinant purified Pol λ after UV-B and high salt stress*

| System | α-Helix  (%) | β-Sheet  (%) | Unordered  (%) | Turn  (%) |
| --- | --- | --- | --- | --- |
| Pol λ Control | 37 ± 4.2 | 22 ± 2.3 | 17 ± 3 | 24 ± 2.8 |
| Pol λ  + 500 mM NaCl | 30 ± 3.4 | 21± 3.1 | 24 ± 2.4 | 25 ± 3.5 |
| Pol λ  + 200 J/m^2^ UV-B | 32 ± 4.1 | 22 ± 1.9 | 25 ± 2.7 | 21 ± 1.8 |
| Pol λ-Del2 Control | 36 ± 2.8 | 19 ± 3.2 | 25 ± 3.1 | 20 ± 3.3 |
| Del2  + 500 mM NaCl | 33 ± 4.1 | 18 ± 4 | 30 ± 2.7 | 19 ± 1.7 |
| Del2  + 200 J/m2 UV-B | 29 ± 3.2 | 18 ± 4.3 | 33 ± 3.4 | 20 ± 2.1 |
| Del3 Control | 30 ± 3.3 | 21 ± 2.5 | 28 ± 4.3 | 21 ± 2.6 |
| Del3  + 500 mM NaCl | 32 ± 3.1 | 19 ± 2 | 27 ± 3.1 | 21 ± 3.1 |
| Del3  + 200 J/m2 UV-B | 31 ± 2.9 | 22 ± 2.7 | 27 ± 2.4 | 20 ± 3.2 |

* Determined from the fitting of the FT-IR data shown in Fig. S8 and S9

# (Errors represent SD from three independent measurements)
